# Impact of selected amino acids of HP0377 (*Helicobacter pylori* thiol oxidoreductase) on its functioning as a CcmG (cytochrome c maturation) protein and Dsb (disulfide bond) isomerase

**DOI:** 10.1371/journal.pone.0195358

**Published:** 2018-04-20

**Authors:** Magdalena Joanna Grzeszczuk, Aleksandra Bąk, Anna Marta Banaś, Paweł Urbanowicz, Stanislaw Dunin-Horkawicz, Artur Gieldon, Cezary Czaplewski, Adam Liwo, Elżbieta K. Jagusztyn-Krynicka

**Affiliations:** 1 Department of Bacterial Genetics, Institute of Microbiology, Faculty of Biology, University of Warsaw, Warsaw, Poland; 2 Structural Bioinformatics Laboratory, Centre of New Technologies, University of Warsaw, Warsaw, Poland; 3 Faculty of Chemistry, University of Gdansk, Gdansk, Poland; University of Nebraska-Lincoln, UNITED STATES

## Abstract

*Helicobacter pylori* HP0377 is a thiol oxidoreductase, a member of the CcmG family involved in cytochrome biogenesis, as previously shown by *in vitro* experiments. In this report, we document that HP0377 also acts *in vivo* in the cytochrome assembly process in *Bacillus subtilis*, where it complements the lack of ResA. However, unlike other characterized proteins in this family, HP0377 is a dithiol reductase and isomerase. We elucidated how the amino acid composition of its active site modulates its functionality. We demonstrated that *cis*-proline (P156) is involved in its interaction with the redox partner (CcdA), as a P156T HP0377 variant is inactive *in vivo* and is present in the oxidized form in *B*. *subtilis*. Furthermore, we showed that engineering the HP0377 active motif by changing CSYC motif into CSYS or SSYC, clearly diminishes two activities (reduction and isomerization) of the protein. Whereas HP0377_CSYA_ is inactive in reduction as well as in isomerization, HP0377_CSYS_ retains reductive activity. Also, replacement of F95 by Q decreases its ability to regenerate scRNase and does not influence the reductive activity of HP0377_CSYS_ towards apocytochrome c. HP0377 is also distinguished from other CcmGs as it forms a 2:1 complex with apocytochrome c. Phylogenetic analyses showed that, although HP0377 is capable of complementing ResA in *Bacillus subtilis*, its thioredoxin domain has a different origin, presumably common to DsbC.

## Introduction

Among the twenty amino acids that are the building blocks of proteins, cysteine residues are the least abundant. On the other hand, cysteine residues are highly conserved in many proteins as they play key role in many physiological processes, such as enzyme activity, metal binding, enzyme stability, and a protein’s cellular localization or regulatory network coordination [1]. The oxidation reaction between two cysteine thiol groups leads to formation of a disulfide bond. For some proteins, the process is essential for their function or stability and also contributes to the pathogenicity of various microorganisms [2, 3]. In gram-negative bacteria, the process of protein oxidative folding takes place in the periplasmic compartment and is catalyzed by a range of proteins, the thiol oxidoreductases of the Dsb (disulfide bond) system. In *Escherichia coli*, where the Dsb system (EcDsb) has been described in detail, its proper functioning is provided by two metabolic pathways: the oxidation pathway, which involves the EcDsbA and EcDsbB redox pair of proteins, and the reduction/isomerization reaction, which relies on the activity of the EcDsbC and EcDsbD redox partners. The first reaction introduces disulfides into polypeptide chains, whereas the second reaction guarantees the rearrangement of non-native disulfides [4, 5].

Thiol oxidoreductases also function in the biogenesis of c-type cytochromes, which occurs in the oxidizing milieu of the bacterial periplasmic compartment. There are five classes of the c-type cytochrome assembly process recognized so far. Of the five, systems I and II mainly act in bacterial cells [6, 7]. System I, which has been characterized in detail for *E*. *coli*, is more complex than system II. It consists of eight proteins (CcmABCDEFGH—cytochrome c maturation) that participate in the process of heme transport and its ligation into reduced apocytochrome. System II, on the other hand, consists of four proteins (CcsA, CcsB, CcdA and CcsX -cytochrome c synthesis) that are needed to ensure cytochrome c assembly [8]. The best characterized system II is that of the gram-positive model organism *Bacillus subtilis*, which consists of four components, ResA (CcsX), ResB (CcsB), ResC (CcsA) and CcdA. ResB and ResC form a complex that is required for heme delivery to the periplasm and its ligation into cytochrome c [9–11]. When the apocytochrome arrives inside the periplasm, a disulfide bond between cysteine residues of its CXXCH motifs is generated by Dsb proteins with oxidizing activity. However, the correct course of the cytochrome c assembly process demands ligation of heme to a reduced thiol of the CXXCH motif of apocytochrome. The specific type of the thiol oxidoreductases displaying reductase activity, called CcmG, which acts in system I as well as in system II, guarantees the reduction of disulfide prior to covalent attachment to heme. CcmGs display a rather low level of homology, but their resolved structures and biochemical properties show multiple similarities. CcmGs are inner-membrane anchored, periplasmic proteins containing a thioredoxin fold with the CXXC motif and a conserved proline (the so-called *cis*-proline loop). Both elements are common for all types of thiol oxidoreductases [12–16]. All CcmGs have a rather low redox potential, ranging from -178 mV for EcCcmG, -213 mV for BjCcmG and -217 mV for PaCcmG, to—256 mV for BsCcmG (ResA) [9, 13, 15, 17]. The reduced state of CcmGs is restored by the action of membrane proteins DsbD or CcdA, which transfer reducing power from cytoplasmic thioredoxin to the periplasm [18, 19]. Both DsbD and CcdA can act in cytochrome c assembly types I and type II, but they are not always interchangeable [20, 21]. The mechanism of electron transport from thioredoxin to their targets by DsbD or CcdA induces several subsequent conformational changes in the electron membrane transporters [18, 19]. Resolving the structure of the DsbDα complex with EcCcmG revealed amino acid residues critical for the interaction between the two proteins [13, 22]. The details of the interaction between CcmGs and CcdAs still remains unknown.

The *H*. *pylori* disulfide bond-forming system is rather simple: this microorganism does not encode the classical DsbA/DsbB, nor DsbC/DsbD, and it possesses only two extracytoplasmic thiol- oxidoreductases, designated HP0231 and HP0377. HP0231 is a dimeric oxidoreductase that is crucial for oxidative protein folding [23–26]. The function of DsbC (the rearrangement of incorrectly paired cysteine residues) may be taken over in *H*. *pylori* by HP0377, another thiol-oxidoreductase. The resolved structure of HP0377 and the location of the *hp0377* gene in the genome indicates that it is a counterpart of CcmG (*cytochrome c maturation*) [16]. However, in contrast to other CcmGs, it does not possess a transmembrane domain in its N-terminus; rather, it is a lipoprotein anchored in the cell membrane. It exists in the cell in the reduced form and possesses a low redox potential (about– 180 mV). Like most CcmGs, it does not reduce insulin, though it untypically possesses disulfide isomerase activity *in vitro*, as it catalyzes the re-folding of scrambled RNase (scRNase). Atypically, HP0377 exists as a mixture of monomeric and dimeric forms, as was demonstrated by exclusion chromatography and glutaraldehyde crosslinking methods. HP0377 is kept in the reduced form by an integral membrane protein, CcdA (HP0265), a shorter version of DsbD. However, HP0377 does not cooperate with EcDsbD. Furthermore, the pKa of the N-terminal cysteine of the CXXC motif of HP0377 is similar to those observed for EcDsbC or EcDsbA, but not to those determined for most CcmGs. Interestingly, HP0377 is an essential protein, and it cooperates with HP0231, as the loss of HP0231 influences the redox state of HP0377. The presented data indicate that the functioning of HP0377 is distinct from other CcmGs [21].

To gain further insight into the *H*. *pylori* Dsb network, we have analyzed HP0377 function in comparison with the well-characterized *Bacillus subtilis* CcmG (ResA). The rationale behind this approach is based on several data. First, both microorganisms use system II for the assembly of cytochrome c. Second, the CcmGs of both bacteria are kept in reduced forms by CcdA. Third, the *B*. *subtilis* ResA is one of the best-characterized CcmGs in terms of structure and mode of action.

## Materials and methods

### Bacterial strains, primers, plasmids, media and growth conditions

Bacterial strains, plasmids and primers used in this study are listed in Tables 1 and 2. The *E*. *coli* strain TG1 was used as a host for the construction and preparation of recombinant plasmids. The *E*. *coli* strain Rosetta (DE3) was used for overexpression experiments. *E*. *coli* strains were grown at 37°C on solid or liquid Luria-Bertani (LB) medium. When needed, media were supplemented with antibiotics at the following concentrations: 100 μg/ml^−1^ ampicillin, 30 μg/ml^−1^ kanamycin and 20 μg/ml^−1^ chloramphenicol. *B*. *subtilis* strains were grown at 30°C in brain heart infusion medium (BHI). Antibiotics, where appropriate, were added to growth media at the following concentrations: 4 μg/ml^−1^ chloramphenicol and 1 μg/ml^−1^ erythromycin. For *B*. *subtilis* strain LUL9 and its derivatives, isopropyl-β-d-thiogalactopyranoside (1 mM) was added to the growth medium. H. *pylori* strain 26695 was grown on Blood Agar base no. 2 (BA) plates (Merck) supplemented with 10% (v/v) horse blood and Oxoid ^™^ *Helicobacter pylori* Selective Supplement (Dent) (ThermoScientific) at 37°C under microaerobic conditions provided by Anoxomat Mark II OP (MARTMicrobiologyB.V).

**Table 1:** Bacterial strains and plasmids used in this study.

Name	Genotype or relevant characteristics	Origin
***Helicobacter pylori* strains**		
26695	*H*. *pylori* wild-type	ATCC
***Escherichia coli* strains**		
BL21 (DE3)	F—*ompT hsdSB*(*rB-mB-*) *gal dcm lon*	Novagen
Rosetta (DE3) LacIq	F—*ompT hsdSB (rB- mB-) gal dcm* pRARE (Cm^r^)	Novagen
TG1	*supE44 hsd*Δ *5 thi* Δ(*lac- proAB*) F’ [*traD36 proAB + lacIq lacZ*ΔM15]	[27]
***Bacillus subtilis* strains**		
168 (1A1)	*B*. *subtilis* wild-type, *trpC2*	BGSC
LUL9	*trpC2 resA*^-^; Em^R^	[9]
BS1	*trpC2 resA*^-^, *amyE*::Pspac-*hp0377*; Em^R^, Cm^R^	This study
BS2	*trpC2 resA*^-^, *amyE*::Pspac-*hp0377*(SSYC); Em^R^, Cm^R^	This study
BS3	*trpC2 resA*^-^, *amyE*::Pspac-*hp0377*(CSYS); Em^R^, Cm^R^	This study
BS4	*trpC2 resA*^-^, *amyE*::Pspac-*hp0377*(SSYS); Em^R^, Cm^R^	This study
BS5	*trpC2 resA*^-^, *amyE*::Pspac-*hp0377*(ASYC); Em^R^, Cm^R^	This study
BS6	*trpC2 resA*^-^, *amyE*::Pspac-*hp0377*(CSYA); Em^R^, Cm^R^	This study
BS7	*trpC2 resA*^-^, *amyE*::Pspac-*hp0377*(ASYA); Em^R^, Cm^R^	This study
BS8	*trpC2 resA*^-^, *amyE*::Pspac-*hp0377*(V*c*P); Em^R^, Cm^R^	This study
BS9	*trpC2 resA*^-^, *amyE*::Pspac-*hp0377*(*c*T); Em^R^, Cm^R^	This study
BS10	*trpC2 resA*^-^, *amyE*::Pspac-*hp0377*(F95E); Em^R^, Cm^R^	This study
BS11	*trpC2 resA*^-^, *amyE*::Pspac-*hp0377*(F95Q); Em^R^, Cm^R^	This study
**General cloning/Plasmid vectors**		
pET28a	Km^r^, IPTG inducible	Novagen
pGEM-T Easy	Ap^r^; LacZa	Promega
pBlusescript SK II	Am^r^, LacZa	Stratagene
**Plasmids for recombinant protein synthesis and purification**		
pUWM518	pET28a/*hp0377*	[21]
pUWM2046	pET28a/*hp0377*_SSYC_	[21]
pUWM2052	pET28a/*hp0377*_ASYC_	This study
pUWM2065	pET28a/*hp0377*_CSYS_	[21]
pUWM2079	pET28a/*hp0377*_ASYA_	This study
pUWM2080	pET28a/*hp0377*_CSYA_	This study
pUWM2083	pET28a/*hp0377*_V*c*P_	This study
pUWM2094	pET28a/*hp0377*_*Tc*T_	This study
pUWM2095	pET28a/*hp0377*_F95A_	This study
pUWM2096	pET28a/*hp0377*_F95E_	This study
pUWM2097	pET28a/*hp0377*_F95Q_	This study
pUWM2090	pET24a/*hp1227*	[21]
pET28a-dsbC	pET28a/*EcdsbC*	J.F. Collet Collection
**Plasmids for *B*. *subtilis* transformation**		
pUWM2157	pVK48/*hp0377*_*TcT*_	This study
pUWM2158	pVK48/*hp0377*_*CSYS*_	This study
pUWM2164	pVK48/wild type *hp0377*	This study
pUWM2165	pVK48/*hp0377*_*SSYC*_	This study
pUWM2166	pVK48/*hp0377*_*SSYS*_	This study
pUWM2167	pVK48/*hp0377*_*CSYA*_	This study
pUWM2168	pVK48/*hp0377*_*ASYA*_	This study
pUWM2169	pVK48/*hp0377*_*ASYC*_	This study
pUWM2170	pVK48/*hp0377*_*VcP*_	This study
pUWM2171	pVK48/*hp0377*_*F95E*_	This study
pUWM2172	pVK48/*hp0377*_*F95Q*_	This study
**Other plasmids**		
pUWM544	pGEM-T easy/*hp0377* without promoter and signal sequence	[21]
pUWM_BS1	pBluescript SK II/ *hp0377* with signal sequence	This study
pVK48	pDH32 derivative; Pspac *lacI*; ApR, Cm^R^	[28]

**Table 2:** Primers used in this study.

Name	Sequence 5’– 3’	Orientation/ Restriction site
Primers used to create vectors for protein purification		
hp377ex1	GAGGCCATGGGCAAATCCAACAATAAAGAC	Fwd/NcoI
hp377ex2	GTGCTCGAGGTTAGACTTGCTTTTAGAAAG	Rev/XhoI
Primers used in site-directed mutagenesis		
hp377_T155V_for	CAAATTTATGCCGTCCAATCCGTCCCTACGATTGTTTTATCCGA	Fwd
hp377_T155V_rev	TCGGATAAAACAATCGTAGGGACGGCATAAATTTG	Rev
hp0377_P156T_for	GGATAAAACAATCGTAGTGGTGGATTGGACGGCAT	Fwd
hp0377_ P156T_rev	ATGCCGTCCAATCCACCACTACGATTGTTTTATCC	Rev
hp0377_F95A_for	TAATGGTTGCTCCTATTGCGAAAGGGCTAAAAAAGATCTCAAAAA	Fwd
hp0377_F95A_rev	CTTTGACATTTTTGAGATCTTTTTTAGCCCTTTCGCAATAGGAGCAA	Rev
hp0377_F95Q_for	TTCTTTGACATTTTTGAGATCTTTTTTTTGCCTTTCGCAATAGGAGC	Fwd
hp0377_F95Q_rev	CGTAATGGTTGCTCCTATTGCGAAAGGCAAAAAAAAGATCTCAAA	Rev
hp0377_F95E_for	TTCTTTGACATTTTTGAGATCTTTTTTTTCCCTTTCGCAATAGGAGC	Fwd
hp0377_F95E_rev	CGTAATGGTTGCTCCTATTGCGAAAGGGAAAAAAAAGATCTCAAA	Rev
hp0377_C89A_for	CTTTTAGTTTTTGGGCGTAATGGTGCCTCCTATTGCGAAAGGTTTAAAAA	Fwd
hp0377_C89A_rev	TTTTTAAACCTTTCGCAATAGGAGGCACCATTACGCCCAAAAACTAAAAG	Rev
hp0377_C92A_for	TTTGGGCGTAATGGTTGCTCCTATGCCGAAAGGTTTAAAAAAGATC	Fwd
hp0377_C92A_rev	GATCTTTTTTAAACCTTTCGGCATAGGAGCAACCATTACGCCCAAA	Rev
hp0377_2Ser_for	AGTTTTTGGCCGTAATGGTTCTTCCTATTCTGAAAGGTTTAAAAAAGATCTC	Fwd
hp0377_2Ser_rev	GAGATCTTTTTTAAACCTTTCAGAATAGGAAGAACCATTACGGCCAAAAACT	Rev
hp0377_2Ala_for	GTTTTTGGCCGTAATGGTGCTTCCTATGCTGAAAGG TTTAAAAAAGATCTCAAAAAT	Fwd
hp0377_2Ala_rev	ATTTTTGAGATCTTTTTTAAACCTTTCAGCATAGGAAGCACCATTACGGCCAAAAAC	Rev
LHP377_RBS_HindIII	GATAAG CTTGAA TGGATC TGCAAT AGGGGG AAGGGG GCAGTG GACAAT GTTTTC ACTTTC TTATG	For
PHP377_RBS_XbaI	GCGTCT AGATTA GGCTTT CCTAGT TAG	Rev

### General DNA manipulations

Standard DNA manipulations were carried out as described in the Sambrook manual [27] or according to the manufacturer’s instructions (A&A Biotechnology, ThermoFisher Scientific). Polymerase chain reactions (PCR) were performed with PrimeStar HS DNA Polymerase (Takara) under standard conditions, according to the manufacturer’s instructions. Synthetic oligonucleotides synthesis and DNA sequencing were performed by Genomed S.A., Warsaw, Poland.

### Cloning and site-directed mutagenesis of full-length HP0377

The wild-type full-length *hp0377* gene was amplified from *H*. *pylori* strain 26695 chromosomal DNA using primers LHP377_RBS_HindIII and PHP377_RBS_XbaI, which introduced a HindIII site at the start of the fragment and an XbaI site at the end. The PCR product was cloned into pBluescript SK II digested with EcoRV, generating pUWM_BS1. Site-directed mutagenesis was carried out using the Quick Change Site-Directed Mutagenesis Kit (Qiagen) according to the manufacturer’s instructions, with pUWM_BS1 as the template to generate mutated *hp0377* genes encoding HP0377 variants. The XbaI/HindIII fragments of pUWM_BS1 and each of the constructed recombinant plasmids were cloned into pVK48 previously cut with the same enzymes, generating plasmids encoding wild-type HP0377 and its mutated variants (Table 1). Sequence analyses confirmed the correct construction of recombinant plasmids, and subsequently, each of pVK48-derived plasmids were used to transform *resA*-defective *B*. *subtilis* strain LUL9. Transformants were selected on BHI plates supplemented with chloramphenicol and IPTG. Plasmid pVK48 allows the introduction of a gene, via a double crossover event, into the chromosome of *B*. *subtilis* at the *amyE* locus, which causes disruption of the *amyE* gene and therefore knocks out amylase activity. Transformants were tested for amylase activity on BHI plates containing 1% (wt/vol) starch using Lugol’s solution. Production of an HP0377 wild type and its mutated versions was confirmed by Western-blot using anti-HP0377 serum. Anti-HP0377 serum was previously produced by rabbit immunization in the Animal Facility, Faculty of Biology, University of Warsaw [21].

### Natural transformation of competent *B*. *subtilis*

The two-step *Bacillus subtilis* transformation procedure was performed as described by Harwood and Cutting [29].

### Protein analysis

Preparation of *E*. *coli* and *B*. *subtilis* protein extracts, SDS-PAGE (sodium dodecyl sulfate polyacrylamide gel electrophoresis) and blotting procedures were performed by standard techniques [27].

#### Overexpression and purification of HP0377 variants, apocytochrome c (HP1227) and EcDsbC for biochemical analysis

To obtain mutated HP0377 proteins for biochemical experiments, a set of recombinant plasmids were constructed. pUWM544 [21], which carries the *hp0377* gene (amino acids 25–221) without its promoter and signal sequence, was used as a starting plasmid for all constructs. Point mutations were generated using the Quick Change Site-Directed Mutagenesis Kit (Qiagen) according to the manufacturer’s instructions, starting with pUWM544 as a template. Correct construction of the plasmids was verified by sequencing. Next, the *hp0377*-modified nucleotide sequences from pUWM544-derived recombinant plasmids were inserted into pET28a. All plasmids carried the HP0377-His_6_ translation fusion. HP0377 and its mutated forms were overexpressed by autoinduction from an *E*. *coli* Rosetta strain and purified using NGC Medium-Pressure Chromatography Systems by Bio-Rad as previously described [21]. HP1227 was overexpressed by IPTG induction and purified from an *E*. *coli* Rosetta strain as previously described [21]. EcDsbC *E*. *coli* protein was overexpressed from *E*.*coli* BL21harboring pET28a/EcDsbC using autoinduction [30].

#### *B*. *subtilis* membrane preparations

Preparation of *B*. *subtilis* membranes was performed as described by Le Brun [28]. *B*. *subtilis* membranes were isolated from cells at stationary phase of growth. Cells were lysed in a reaction buffer and centrifuged to remove unbroken cells. The membranes were recovered from the supernatant by centrifugation, were resuspended in 20 mM MOPS pH 7.4 and were stored at -80° C.

### Biochemical assays

#### In vivo redox state of *HP0377* and its mutated versions in *B*. *subtilis*

The redox states of HP0377 wild type and its mutated version were visualized by alkylating the free cysteine residues using 4-acetamido-4′-maleimidylstilbene-2,2′-disulfonic acid (AMS, Invitrogen). This agent modifies only covalently free thiols, resulting in an increase of a protein molecular mass of 490 Da/every—SH group [31]. Briefly, bacteria were harvested from BHI plates after overnight incubation in 37°C. Samples were standardized using the OD_600_ of the culture, and ice-cold trichloroacetic acid (TCA, final concentration 10% v/v) was immediately added to the culture. Whole-cell proteins were precipitated and collected by centrifugation, washed with ice-cold acetone, and then dissolved in a reaction buffer containing 10 mM AMS by agitation for 60 min at 37 C, and then resolved by 14% SDS-PAGE without reducing agent. HP0377 was then detected by an immunoblot analysis using an anti-HP0377 antibody. As controls, we used samples treated with DTT before precipitation of the proteins with TCA.

#### Determination of isomerase activity, refolding of scrambled RNase A

*In vitro* refolding of scrambled RNase A was performed for HP0377 variants and EcDsbC as described earlier [21]. HP0377 variants were reduced with DTT and mixed with previously prepared scrambled RNase. The redox state of the thiols was confirmed by Ellman’s assay. RNase A activity was measured by analyzing the cleavage of cCMP. EcDsbC was used as a positive control. Three independent experiments were performed.

#### Determination of HP0377’s ability to reduce apocytochrome c *in vitro*

Oxidized apocytochrome (HP1227) was prepared by incubating purified HP1227 with a 100 mM oxidized glutathione for 1 h at 37°C. Reduced HP0377 variants were prepared by incubating the purified HP0377 variants with 100 mM DTT for 1 h at 37°C. Excess DTT and oxidized glutathione were removed using desalting columns that were previously equilibrated with 20 mM Tris HCl pH 7.9, 150 mM NaCl. Oxidized apocytochrome c in 20 mM Tris–HCl pH 7.9, 150 mM NaCl was incubated for 3 h at 37°C in the presence of a twofold excess of HP0377_red_ variants. Subsequently, the proteins were precipitated with trichloroacetic acid [final concentration 10% (w/v)], washed three times with ice-cold acetone and then resuspended in 100 μl of a reaction buffer consisting of 50 mM Tris–HCl pH 6.8, 2% (w/v) SDS, 20 mM AMS. AMS-treated protein samples were separated by nonreducing SDS–PAGE and visualized by Coomassie staining. The oxidized and reduced forms of HP0377, as well as reduced apocytochrome c, were similarly treated with AMS before examination by SDS–PAGE under nonreducing conditions.

#### Determination of the oligomeric state of HP0377 versions using glutaraldehyde

Crosslinking of polypeptide chains with glutaraldehyde was performed essentially as previously described, by incubating HP0377 variants with different concentration of glutaraldehyde [21].

### Assays for cytochromes *c*

#### TMPD staining for cytochrome *c* oxidase activity

*B*. *subtilis* strains deficient in cytochrome *c* oxidase *caa*_3_ were identified by testing for *N*,*N*,*N*′,*N*′-tetramethyl-*p*-phenylenediamine (TMPD) oxidation, as described by Le Brun et al. [28]. BHI plates with overnight cultures of *B*. *subtilis* transformants harboring wild type and mutated versions of *hp0377* were stored at -20°C for 12 min before coating with TMPD stain (0.8% Triton X-100 in potassium phosphate pH 7, 0.4% sodium deoxycholate, 20% EtOH, 0.6% w/v agar, 0.2% w/v TMPD). Colonies that turned blue demonstrated cytochrome *c* oxidase activity.

#### Cytochrome *c* oxidase assay

The activity of cytochrome *c* oxidase *caa*_3_ was measured quantitatively by using a cytochrome *c* oxidase activity assay as described by Hodson et al. [32]. Briefly, samples of reduced horse heart cytochrome *c* in 20 mM 3-morpholinopropanesulfonate (MOPS) (pH 7.4) were mixed with purified membrane preparations. Changes in absorbance at 540 and 550 nm were measured every 15 s. Differences in absorbance (*A*_550_ − *A*_540_) were plotted against time. The rate in absorbance per minute was converted to mM cytochrome *c* oxidized per minute (d[cyt]ox/dt) using the equation:

$$\frac{d[cyt]ox}{dt}=\frac{dA}{dt}\times \frac{\left[cyt\right]}{\Delta At}$$

where [cyt] is the concentration of cytochrome *c* used and ΔAt is the total absorbance change going from fully reduced to fully oxidised cytochrome *c*.

#### Heme staining

Heme staining was performed as described by Thony Meyer et al. [33]. Proteins of membranes isolated from *B*. *subtilis* strains were separated by SDS-PAGE. Next, the gel was soaked in 10% trichloroacetic acid (TCA), washed twice in water, and incubated in staining solution (1 mg/ml *o*-dianisidine-HCl, 0.1 M trisodium-citrate pH 4.4, 0.7% w/v H_2_O_2_) at room temperature. The reaction was stopped by washing the gel in water. Heme containing proteins were visible as the *o*-dianisidine turns green when oxidised by heme/H_2_O_2_.

### Complex formation

#### Trapping and purification of the HP0377-apocytochrome complex

HP0377_CSYA_ was overexpressed by overnight autoinduction of an *E*. *coli* Rosetta strain carrying pUWM2080. HP1227 was overexpressed by 6 h induction with 1 mM IPTG (isopropyl β-D-1-thiogalactopyranoside), starting at OD600 ~ 0.6 for an *E*. *coli* Rosetta strain carrying pUWM2090. Both cultures (400 ml of *E*.*coli*/pUWM2080 and 10 l of *E*. *coli*/pUWM2090) were centrifuged and the cell pellets were suspended together in 50 mM sodium phosphate, pH 8.0, 300 mM NaCl, 10 mM imidazole (about 10 ml of the buffer/gram of wet cell mass). The disproportionate volumes of the cultures used for the experiment was due to weak expression of HP1227. Cells were disrupted by ultrasonication. The cell lysate was centrifuged to remove unbroken bacterial cells, and the resulting supernatant, after overnight incubation at 37°C, was applied onto Bio-Scale Mini Profinity IMAC Cartridges (Bio-Rad) containing Ni-charged resin. Proteins were eluted with an imidazole gradient, using the NGC chromatography system (Bio-Rad). After purification, protein-containing fractions were loaded onto ENrich^™^ SEC70 Column (Bio-Rad) and eluted with 20 mM Tris pH 8, 150 mM NaCl. After size exclusion chromatography, fractions containing HP0377 complex with apocytochrome c were analyzed by SDS-PAGE, with or without addition of DTT.

### Other methods

#### Bioinformatics analyses

HHpred [34] server was used to identify the thioredoxin domain in the HP0377 protein sequence. The sequence of the HP0377 thioredoxin domain was used as a query to search an nr90 database (NCBI non-redundant protein sequence database filtered at 90% sequence identity) with PSI-BLAST (5 iterations, default parameters). Matches with coverage below 85% or an e-value larger than 1e-5 were discarded and the remaining 68,088 sequences were filtered to 70% identity with CD-HIT [35] and clustered using CLANS [36]. The CLANS cluster map was manually analyzed and various cut-off values and four clusters were defined. Sequences from each cluster were aligned with Clustal Omega [37] and filtered with HHfilter to obtain representative sequences. The representative sequences from of all clusters were collected together and aligned with PROMALS [38]. The alignment of 161 sequences was subsequently refined with MUSCLE [39] and gap-rich columns were removed. A phylogenetic tree was calculated with FastTree [40] and visualized with EvolView [41]. The tree was rooted using a Thioredoxin-like clade as an outgroup.

#### Analysis of the fluctuations of HP0377 and its mutated versions

**Fig 1:**
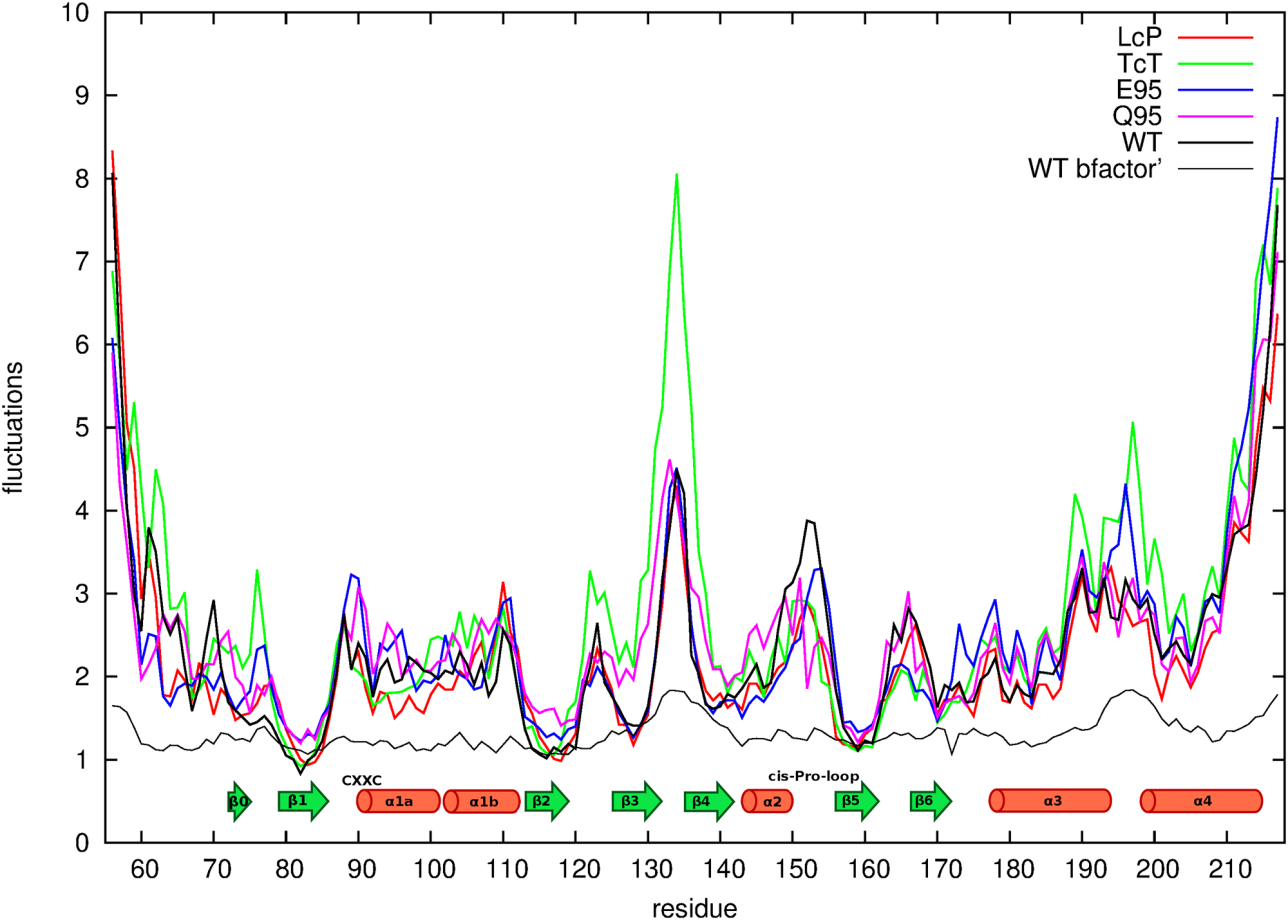
Plots of the fluctuations of the α-carbon atoms as obtained by the UNRES simulation. The secondary-structure elements are shown above the abscissa. The black, thin line corresponds to the β factors as obtained from the HP0377 (PDB: 4FYC) structure. The simulation results are marked as follows: wild-type HP0377 (black “WT”); CSYC, LcP, (red, “LcP”); CSYC, TcT, (green, “TcT”). E95 and Q95 are colored blue and purple, respectively.

To determine the effect of mutations on the dynamics of studied proteins, short canonical molecular-dynamics simulations with a coarse-grained UNRES force field were run for the wild-type protein and the mutants [42]. The simulations were run at T = 250 K. The duration of simulation was 1 ns and the temperature was controlled by the Langevin thermostat. The last version of the UNRES force field, calibrated with 7 small proteins, was used [43]. Use of a coarse-grained force field was motivated by the ability of the coarse-grained approach to simulate conformational changes in a shorter time. The fluctuations were defined as root-means-square deviations of the positions of the a-carbon-atom coordinates. The plots of the fluctuations in residue indices are shown in Fig 1. For comparison, the fluctuations calculated from the crystallographic β-factor are also shown in the Fig 1; they are related to the β-factor by equation.

$$\sigma_{i}=\sqrt{\frac{3}{8\pi }}\beta_{i}$$

where σ is the magnitude of the fluctuations for the ith α-carbon atom, and β_*i*_ is the experimental β-factor for that atom. Due to the crowded environment of a molecule in the crystal phase, the fluctuations calculated from the β-factors are several times smaller than those computed from MD simulations. However, the shape the fluctuations plot obtained from MD should generally resemble (except for the N- and the C-terminus) the experimental plots. Consequently, it is legitimate to use the fluctuations plots in order to assess the effect of mutations on protein dynamics.

## Results

### 1. HP0377 acts as an apocytochrome c reductase in *Bacillus subtilis*

We have previously shown that both HP0377 (CcmG) and HP0378 (CcsAB), which is involved in heme transport and its ligation to apocytochrome c, provide essential functions for *H*. *pylori*. This makes analysis of HP0377 activity in its native host impossible. Thus, we decided to examine HP0377 functioning *in vivo* in *B*. *subtilis*, which like *H*. *pylori* uses a system II cytochrome c assembly process. Furthermore, structural superposition of ResA and HP0377 revealed that both structures are similar, though not identical, and both have structural hallmarks characteristic for canonical CcmGs (S1 and S2 Figs).

**Fig 2:**
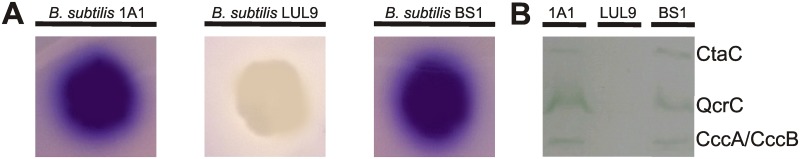
HP0377 restores wild type phenotype in *Bacillus subtilis ΔresA* strain. (**A**) TMPD staining of bacterial colonies; a dark pigmentation indicates cytochrome *c* oxidase activity. Strain 1A1 is a wild-type *B*. *subtilis*, LUL9 is a ResA-deficient strain, BS1 is a derivative of LUL9 harboring wild-type *hp0377* integrated at the *amyE* locus, (**B**) SDS/PAGE examination of *B*. *subtilis* membranes stained for covalently bound heme. Cytochromes indicated are CtaC (subunit II of cytochrome *caa*_*3*_), QcrC (subunit *c* of the cytochrome *bc* complex) and CccA/B (cytochrome *c*_550_ and cytochrome *c*_551_ which are not resolved).

The PVK48 plasmid, which allows the introduction of a gene of interest into the chromosome of *B*. *subtilis* at the *amyE* locus, was a starting point for HP0377 functional analysis in *B*. *subtilis* cells. pUWM2164 was constructed and introduced by transformation into *B*. *subtilis* LUL9, a derivative of the *B*. *subtilis* wild type strain in which the native *resA* gene is disrupted [9]. Details of construction of the recombinant *B*. *subtilis* containing the *hp0377* gene integrated into chromosome are given in materials and methods section. The production of HP0377 in B. *subtilis* cells was verified by Western blot analysis using specific rabbit anti-HP0377 antibody (S3 Fig). As the first step in testing activity of HP0377 in *B*. *subtilis*, we used the colony staining test with the cytochrome caa_3_ (cytochrome c oxidase)-specific artificial substrate—TMPD. This test showed that HP0377, in agreement with expectations, efficiently substituted for the lack of ResA (Fig 2A). Next, to more precisely quantify the level of its activity, a spectroscopic cytochrome c oxidase assay was carried out. We found that complementation of the *B*. *subtilis* LUL9 strain with *hp0377* restored a wild-type phenotype (Table 3). In order to gain insight into the process, the content of four cytochromes c in the *B*. *subtilis* cell membrane was also evaluated by a heme-staining method. As shown in Fig 2B, the four *B*. *subtilis* c-type cytochromes in the LUL9 strain complemented with HP0377 were present in the membrane, essentially at the level characteristic for wild type *B*. *subtilis*. All together, the tests revealed that HP0377 was involved in the cytochromes c maturation process in this heterologous host and provided activity at levels identical to that of ResA.

**Table 3:** Cytochrome *c* oxidation activities of membranes from different *B*. *subtilis* strains.

Strain	HP0377 variants	Oxidation activity (nM min^-1^ mg protein^-1^)
**1A1**	**-**	**85±5**
**LUL9**	**-**	**3±2**
**BS1**	**Wild type**	**88±7**
**BS2**	**SSYC**	**79±7**
**BS3**	**CSYS**	**84±3**
**BS4**	**SSYS**	**7±3**
**BS5**	**ASYC**	**8±4**
**BS6**	**CSYA**	**6±2**
**BS7**	**ASYA**	**4±1**
**BS8**	**V*c*P**	**83±4**
**BS9**	**T*c*T**	**19±7**
**BS10**	**F95E**	**82±3**
**BS11**	**F95Q**	**84±6**

### 2. N- and C-fragments of HP0377 are not essential for its function

**Fig 3:**
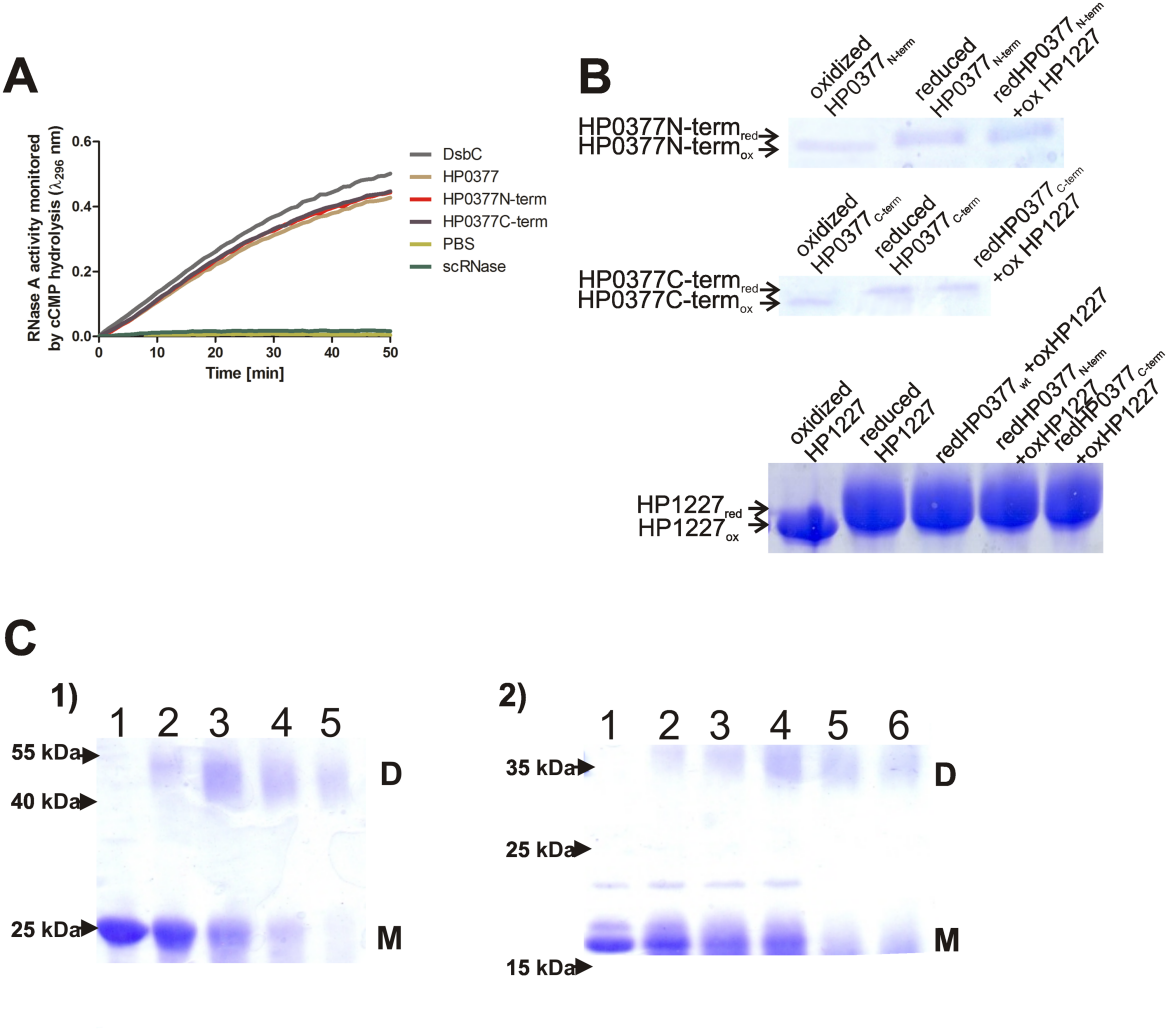
HP0377 truncated variants act as wild type HP0377. **(A)*In vitro* isomerase activity assay**. The reaction contained 40 μM scrambled RNase in 200 mM potassium phosphate buffer, pH 7.0, 2 mM EDTA, 20 μM DTT, and 9 mM cCMP. The reaction was performed in the absence or presence of 20 μM EcDsbC, 20 μM HP0377 and its variants. The cleavage of cCMP by refolded RNase was monitored continuously at 296 nm. The changes in the absorbance at 296 nm as a function of time are presented. Three independent experiments were performed. **(B) *In vitro* reductase assay of HP0377 variants towards apocytochrome c (HP1227)**. Two different SDS PAGEs were run to better visualize the shift between oxidized and reduced forms of proteins. Different redox forms were detected by nonreducing SDS-PAGE after AMS treatment, which results in an increase in the molecular mass of reduced proteins by about 0.5 kDa per thiol group. **(C) Glutaraldehyde crosslinking of truncated versions of HP0377. 1) N-terminal-shortened HP0377; 2) C-terminal-truncated HP0377**. Purified HP0377 truncated versions at 2.5 mg/ml were cross-linked in the presence of different concentration of glutaraldehyde: 1) purified HP0377 protein, 2) 0.001%, 3) 0.005%, 4) 0.01%, 5) 0.05%, 6) 0.1% glutaraldehyde. M—monomers, D—dimers.

Although, the HP0377 architecture resembles those of other CcmGs, there are noticeable differences, which, among others, concern the C-termini of the compared proteins (S4 Fig). HP0377 possesses an atypical extension at the C-terminus. So, we asked whether C-terminus fragments of HP0377 were involved in its atypical features, mainly its ability to isomerize scRNase. Two versions of the oxidoreductase were constructed. The first contained amino acids 25–187 of the native HP0377, and the second contained amino acids 33–221 of the native HP0377. The rationale behind construction the 33–221 HP0377 version was to examine the HP0377 variant that was previously analyzed by Yoon et al. [16]. Both of the HP0377 truncated forms were purified from *E*. *coli* and analyzed in terms of their capability to reduce the oxidized form of apocytochrome c and isomerize scRNase (Fig 3A and 3B). We also checked the ability of the two truncated HP0377 versions to oligomerize, using the glutaraldehyde crosslinking strategy (Fig 3C). We found that the missing fragments of the proteins were not required for scRNase isomerization or apocytochrome reduction. Additionally, both HP0377 versions analyzed, similar to the previously analyzed 25–221 HP0377 variant, generated dimeric forms with molecular masses of 46 kDa (33–221 variant of HP0377) and 34 kDa (25–187 variant of HP0377), after exposure to glutaraldehyde.

### 3. Changing the amino acid sequence of the HP0377 active site influences its activity *in vitro* and *in vivo*

**Fig 4:**
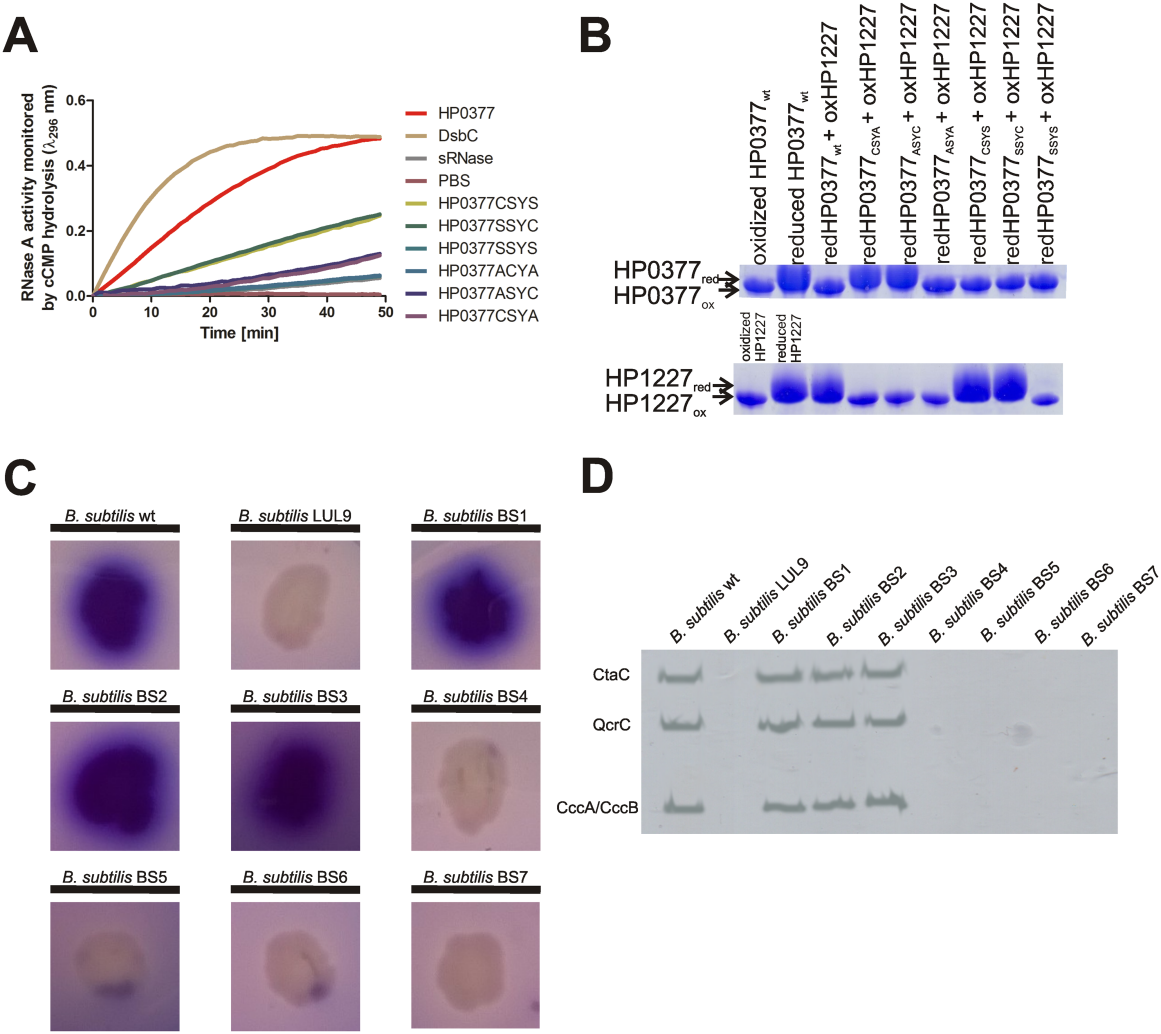
*In vivo* and *in vitro* properties of HP0377 with engineered catalytic CSYC motifs. **(A) *In vitro* isomerase activity assay**. The reaction contained 40 μM scrambled RNase in 200 mM potassium phosphate buffer, pH 7.0, 2mM EDTA, 20 μM DTT, and 9mM cCMP. The reaction was performed in the absence or presence of 20 μM EcDsbC, 20 μM HP0377 and its variants. The cleavage of cCMP by refolded RNase was monitored continuously at 296 nm. The changes in the absorbance at 296 nm as a function of time are presented. Three independent experiments were performed. **(B) *In vitro* reductase assay of HP0377 variants towards apocytochrome c (HP1227)**. Two different SDS PAGEs were run to better visualize the shift between oxidized and reduced forms of proteins. Different redox forms were detected by nonreducing SDS-PAGE after AMS treatment, which results in an increase in the molecular mass of reduced proteins by about 0.5 kDa per thiol group. **(C) TMPD stains of *B*. *subtilis* wild type, ResA-deficient and *B*. *subtilis* carrying HP0377 active site variant strains**. *B*. *subtilis* colonies made up of cells with an active cytochrome *c* oxidase are stained blue. BS1 is a derivative of *B*.*subtilis resA* complemented with HP0377 wt, BS2 with HP0377 (SSYC), BS3 with HP0377 (CSYS); BS4 with HP0377 (SSYS), BS5 with HP0377 (ASYC), BS6 with HP0377 (CSYA); BS7 with HP0377 (ASYA). **(D) Heme staining of membranes from *B*. *subtilis* wild type, ResA-deficient and *B*. *subtilis* carrying HP0377 mutated variants**.

Despite sharing several structural and biochemical properties with other CcmGs characterized so far, the multiple sequence alignment of chosen members of the CcmGs family revealed several potentially significant differences between HP0377 and other CcmGs (S4 Fig). To elucidate the role of selected amino acids in HP0377 functioning *in vivo* and to determine whether the noticeable sequence alignment differences are reflected in biochemical features of HP0377, we therefore generated a set of mutated versions of the HP0377 thiol oxidoreductase. First, we constructed several HP0377 variants with the cysteine residues of the CSYC motif replaced by alanine or serine, singly or doubly. All mutated variants of HP0377 were subjected to biochemical analysis *in vitro*, and their functions were investigated *in vivo* in *Bacillus subtilis* cells. The *in vitro* test used to specify the redox state of apocytochrome c and HP0377 is based on AMS reagent, which interacts with the thiol group of cysteine residues, leading an increase of the molecular mass of the protein by 490 Da for each free thiol group. Note that two of the HP0377 variants with changed CSYC active motifs (the ASYA and SSYS motifs) do not contain cysteine residues and thus cannot react with AMS; they exhibited the same mobility as the oxidized form of HP0377. Four other HP0377 variants (ASYC, CSYA, SSYC and CSYS motifs) react with only one AMS molecule, and therefore have faster electrophoretic mobility compared to the wild type reduced form of HP0377 treated with AMS. Of the six variants, four (containing the CSYA, ASYC, ASYA and SSYS motifs) were inactive, both *in vivo* and *in vitro*, in apocytochrome c reduction and the scRNase isomerization test (Fig 4). Remarkably, the other two HP0377 variants, which had one cysteine residue of the CSYC motif changed to serine, were functional in the process of apocytochrome c reduction, both *in vivo* and *in vitro*, but were severely impaired in their ability to regenerate scRNase *in vitro*. Therefore, we conclude that replacing one cysteine residue of the CSYC motif with serine still allows HP0377 functioning in reduction, but not in shuffling non-native disulfide bonds, at least *in vitro*.

**Fig 5:**
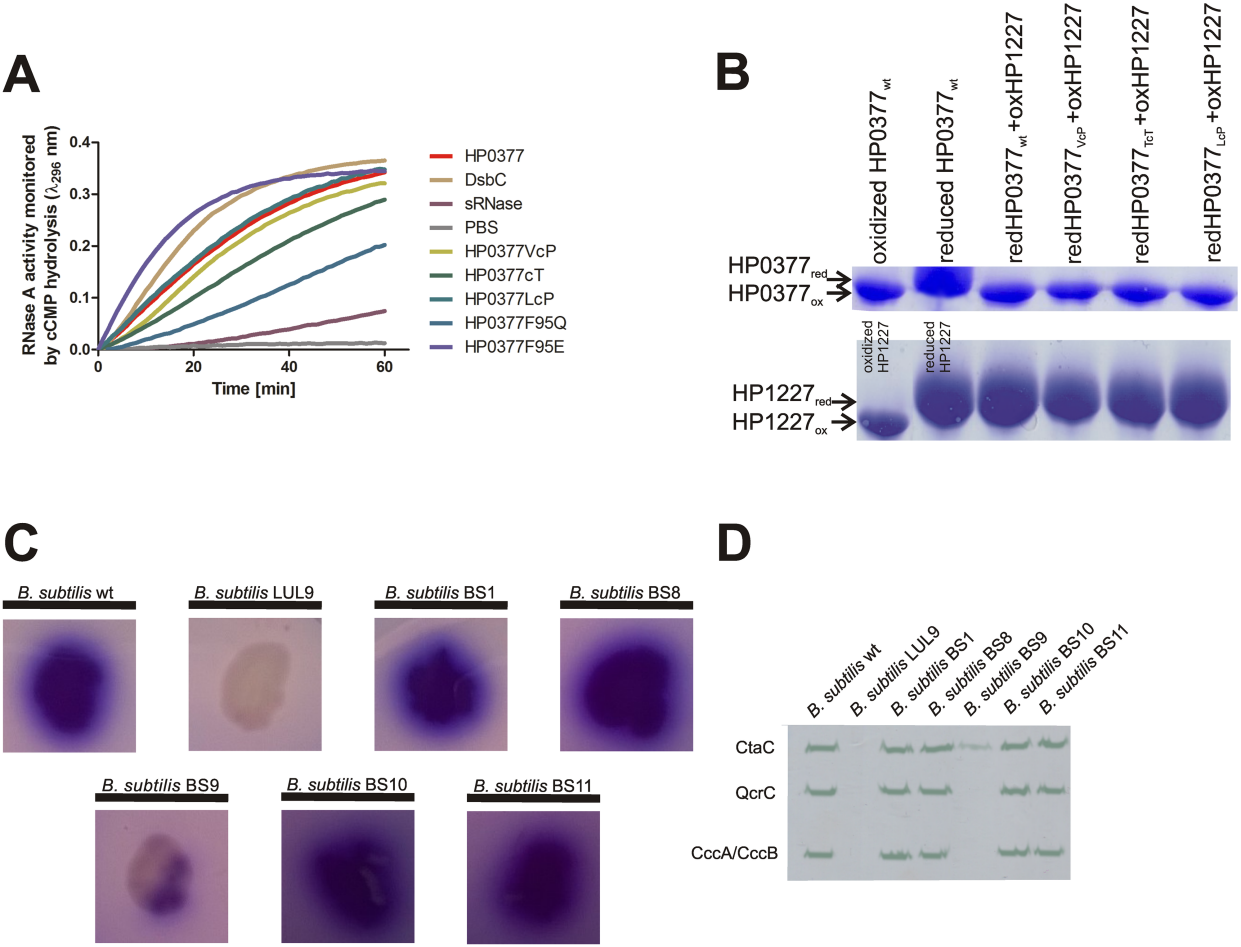
*In vivo* and *in vitro* properties of HP0377 with engineered *cis*-proline loops and F95 variants. **(A) *In vitro* isomerase activity assay**. The reaction contained 40 μM scrambled RNase in 200 mM potassium phosphate buffer, pH 7.0, 2mM EDTA, 20 μM DTT, and 9mM cCMP. The reaction was performed in the absence or presence of 20 μM EcDsbC, 20 μM HP0377 and its variants. The cleavage of cCMP by refolded RNase was monitored continuously at 296 nm. Changes in the absorbance at 296 nm as a function of time are presented. Three independent experiments were performed. **(B) *In vitro* reductase assay of HP0377 variants towards apocytochrome c (HP1227)**. Two different SDS PAGEs were run to better visualize the shift between oxidized and reduced forms of proteins. Different redox forms were detected by nonreducing SDS-PAGE after AMS treatment, which results in an increase in the molecular mass of reduced proteins by about 0.5 kDa per thiol group. **(C) TMPD stains of *B*. *subtilis* wild type, ResA-deficient and HP0377 variant strains**. *B*. *subtilis* colonies made up of cells with an active cytochrome *c* oxidase are stain blue. BS1 is a derivative of *B*.*subtilis resA* complemented with HP0377 wt, BS8 with HP0377 (V*c*P), BS9 with HP0377 (T*c*T); BS10 with HP0377 (E95), BS11 with HP0377 (Q95). **(D) Heme staining of membranes from *B*. *subtilis* wild type, ResA-deficient and HP0377 variant strains**.

**Fig 6:**
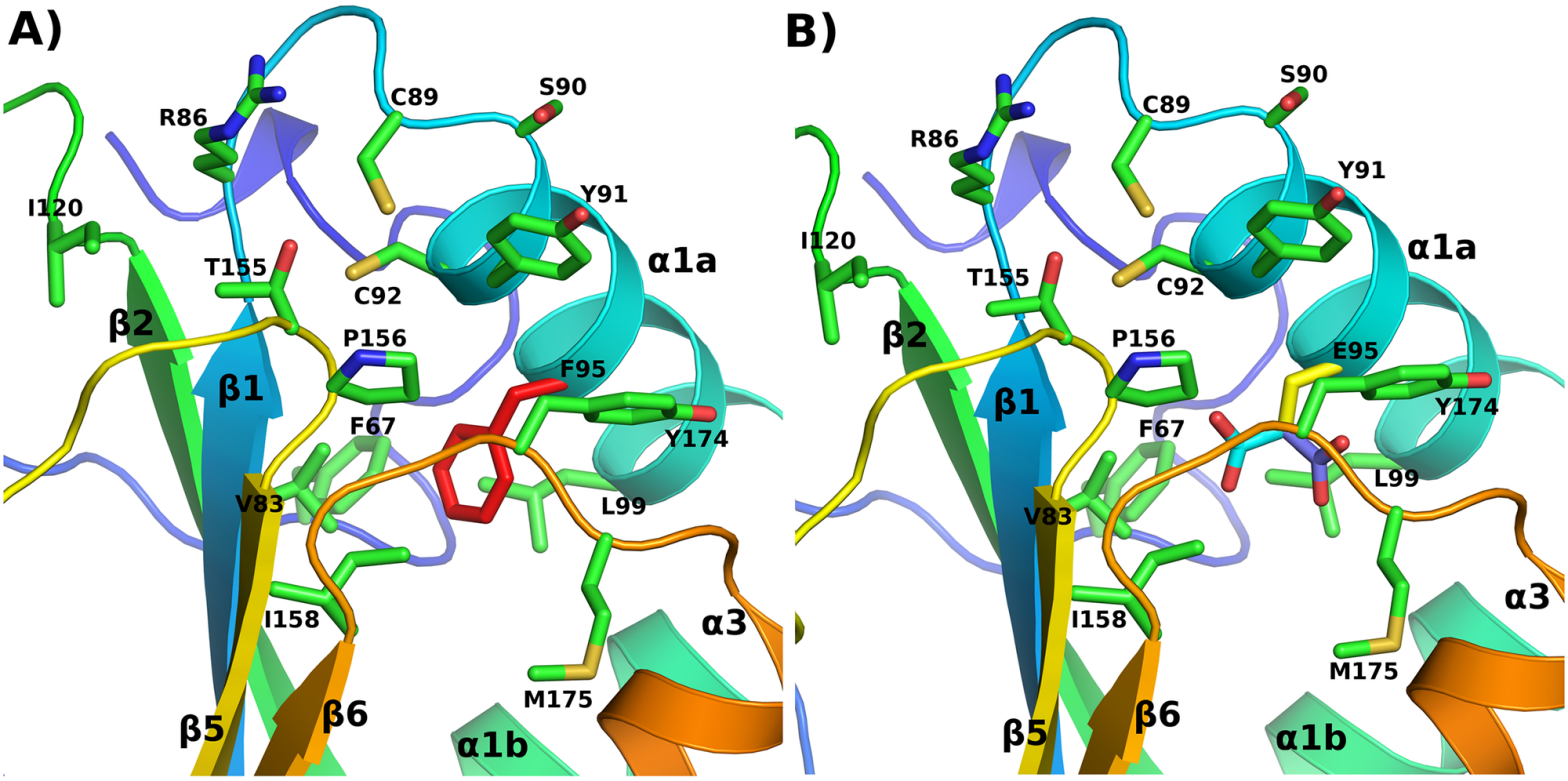
Visualization of the active site and the interface of β1, β5 and α1 of (A) HP0377 (PDB: 4FYC), (B) its F95/E mutant. The position of F95 is marked red, two probable rotamers of E95 are marked purple.

The catalytic active site of HP0377 (CSYC) resembles the active site of the disulfide isomerases EcDsbC (CGYC) or CcScsC (CGYC), rather than the active sites of many other CcmGs that contain at least one proline residue in the internal dipeptide of CXXC motif. Table 4 presents the main biochemical features of well-characterized CcmGs. HP0377 is also distinguished from other CcmGs by its *cis*-proline loop, which is an important element of the active sites of thiol oxidoreductases [44]. Most of the CcmGs contain a small amino acid with a hydrophobic side chain (alanine, valine or leucine) as the amino acid that precedes *cis*-proline. In contrast, the *cis*-proline loop of HP0377 (T*c*P) contains the hydrophilic threonine as the amino acid that precedes *cis*-proline, a characteristic conserved among disulfide isomerases. Thus, we asked the question whether the composition of the active site has an impact on HP0377 functioning. Three variants of HP0377 were generated by site directed mutagenesis. The first and the second have an own catalytic motif (CSYC) paired with L*c*P or V*c*P, instead of T*c*P, and the third possesses a native CSYC motif combined with T*c*T (CSYC/T*c*T). The LcP motif is present in *B*. *subtilis* ResA, whereas the V*c*P motif is characteristic for monomeric Dsb proteins with oxidizing activity. Lewin et al. documented that the glutamic acid (E80), located in the vicinity of the binding cavity of *B*. *subtilis* ResA, plays a key role in its interaction with substrate. This amino acid is conserved among many extracytoplasmic thiol oxidoreductases in the CcmG family but not among cytoplasmic thioredoxins [10]. HP0377 atypically possesses phenylalanine (F) in this position. This prompted us to investigate its role in substrate binding, and we constructed two extra mutated variants of HP0377 in which F95 was changed into Q or E, respectively. All versions of HP0377 lacking signal sequences were purified from *E*. *coli* cells by affinity chromatography and analyzed in respect of their biochemical features (isomerization of scRNase and ability to reduced oxidized apocytochrome c). Additionally, the *in vivo* activity of some was also investigated in *B*. *subtilis* cells. The presence of various HP0377 variants in *B*. *subtilis* was verified by Western blot using specific rabbit anti HP0377 serum (S2B Fig). The F95E HP0377 variant showed an isomerization activity even higher than EcDsbC, whereas changing F95 to Q significantly decreased its ability to isomerize scRNase. The F95E and F95Q substitutions, however, did not affect the cytochrome maturation process (Fig 5, Table 3). Thus, we showed that the amino acid in this position is an important factor that modulates the reactivity of the protein, at least when scRNase is used as a substrate. Fig 6 presents the visualization of the active site and the interface of β1, β5 and α1 of HP0377 and its F95E mutant. The rotamer analysis of the F95E mutant shows two putative positions of E95 (Fig 6B) (similar side chain positions were observed in the case of the Q95 variant, not shown). In the first case, E95 is directed towards the loop connecting β6 and α3 and makes a hydrogen bond interaction with the backbone of M175. In the second case, E95 is directed towards P156. F95 is located in the center of the hydrophobic nest containing F67, V83 L99 and I158 (Fig 6A). It may be that the role of this residue is to maintain stability of the protein by preserving the mutual orientation of β1, β5 and α1 from the thioredoxin fold. The replacement of F95 by E/Q lowers this ability. However, E95 is able to form a hydrogen bond with the M175 backbone. Therefore, the formation of this hydrogen bond can also stabilize the protein structure. This is not possible with F95/Q mutant. If we compare the interaction energy for the following pairs E-P and Q-P [45], we can see that E-P is only 1 kcal/mol lower than Q-P. A 1 kcal/mol decrease does not explain the greatly diminished isomerization activity of the F95Q HP0377 enzyme observed with scRNA. However, with the presence of the acidic residue located in the vicinity of the cysteine, an oxidation reaction between two cysteine thiol groups is much easier, compared to a variant with the amide group (Q95).

**Table 4:** Characteristics of different CcmG proteins.

Organism	protein	Redox potential [mV]	Redox partner [46]	pKa	Cytochrome c biogenesis type	CXXC motif	Cis-proline motif	Fingerprint region GVXGXPETF
*Bacillus subtilis*	ResA (CcmG)	-256 [47]	CcdA	8.8	II	CEPC	LcP	(-)
*Helicobacter pylori*	HP0377	-180 [16] -176 [21]	CcdA	3.5	II	CSYC	TcP	(-)
*Mycobacterium tuberculosis*	Rv3673c (CcsX)	nt	CcdA	nt	II	CGPC	IcP	(-)
*Bradyrhizobium japonicum*	CcmG (CycY)	-213 [17]	CcdA ScsBα	nt	I	CVPC	VcP	GVYGVPETF
*Bradyrhizobium japonicum*	TlpA	-256 [48]	CcdA ScsBα	nt	*	CVPC	McP	GVYGVPETF
*Paracoccus denitrificans*	CcmG	nt	CcdA ScsBα	nt	I	CPPC	PcP	GVTAPPETF
*Rhodobacter capsulatus/spheroides*	HelX/CcmG	nt	CcdA ScsBα	nt	I	CAPC	VcP	GVAGVPETF
*Bordetella pertussis*	CcsX (CcmG)	nt	DsbD	nt	II	CAPC	VcP	GVYGVPETF
*Campylobacter jejuni*	Cj1106 ** CcsX—ResA like	nt	DsbD	nt	II	CTPC	??	(-)
	Cj12072 CcsX-ResA like	nt	DsbD	nt	II	CTPC	IcP	GVNGIPTMF
*Escherichia coli*	CcmG	-178 [13]	DsbD	6.8	I	CPTC	AcP	GVYGAPETF
*Pseudomonas aeruginosa PAO1*	CcmG	-213 [15]	DsbD	6.13	I	CPSC	VcP	GVYCAPETY

*TlpA of Bradyrhizobium japonicum acts as a reductant for cooper metallochaperon ScoI and for CoxB [49]

**Cj1106 was classified as CcsX/ResA-like protein. However its role in the cytochrome biogenesis is not completely clear as it is involved only in the assembly process of certain type cytochromes [50]

Underlined fingerprinting regions are similar but not identical.

**Fig 7:**
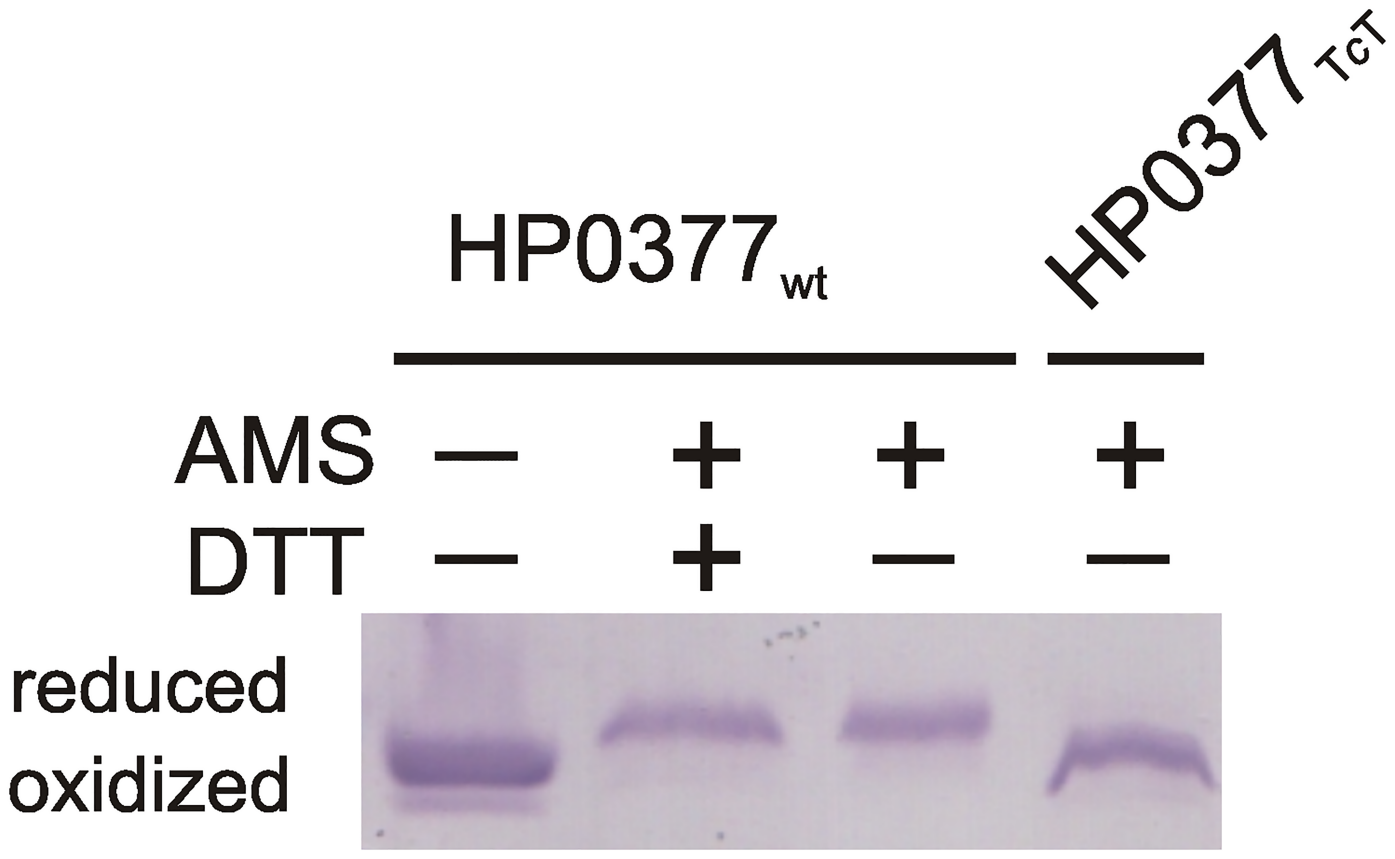
HP0377_TcT_ is present in the oxidized form in *B*. *subtilis* BS9 cells. Bacterial cultures were treated with 10% TCA, followed by alkylation of—SH groups of cysteine with AMS. Cellular proteins were separated by 14% SDS-PAGE under non-reducing conditions. HP0377 reduced form (red; DTT treated, modified with AMS) and the oxidized form (ox; non-modified with AMS) were used as controls. HP0377s were visualized by Western blot analysis using rabbit specific antibodies against HP0377. Each lane contains proteins isolated from the same amount of bacterial cells.

Replacement of proline by threonine in the *cis*-proline loop (P156T) significantly decreased the ability of the HP0377 to reduce apocytochrome c *in vivo* (Fig 5C and 5D, Table 3), but it did not influence its reactivity in *in vitro* tests (Fig 5A and 5B), indicating the crucial role of this amino acid in the interaction with redox partner CcdA. The HP0377 used for in *vitro* tests was present in the reduced form, as it was treated with DTT. To confirm the role of the P156 residue in the interaction with its redox partner, the redox state of the P156T HP0377 variant was also evaluated in *B*. *subtilis* by AMS-trapping technique. We found that, in contrast to HP0377 wild type, which exists in the reduced state, HP0377 with the T*c*T motif is present in the oxidized form (Fig 7). Changing the T*c*P proline loop into L*c*P, as found in ResA, or into V*c*P had no impact on HP0377 functioning in the tests we used (Fig 5). To determine the effect of mutations on the dynamics of the HP0377 protein, short canonical molecular-dynamics simulations with the coarse-grained UNRES force field were run for the wild type protein and for the mutants. As can be seen from Fig 1, all residues except the C-terminal part of α3, and all secondary-structure elements from thioredoxin fold remain stable, with fluctuations less than 2Å. One of the most important questions was how the mutation T*c*P to T*c*T in the *cis*-proline loop influenced the HP0377 structure. In this simulation, we found three regions with an increased mobility in comparison to the wild-type protein, namely: the loops connecting β2—β3, β3—β4 and α3—α4. Surprisingly, we did not observe any increased mobility in the *cis*-proline loop itself. Only one of the fragments under investigation (the loop connecting β2 and β3) is located in the vicinity of the *cis*-proline loop and the CXXC motif. It can, therefore, be expected that this fragment plays a role in the binding of the redox partner. The other two fragments are located on the other side of the protein. It may be that the T*c*T mutation results in a propagation of motions on to other structural fragments, and as a consequence, the mutant is not active. On the other hand, the analysis of fluctuations did not provide any reasonable explanation as to why the F95E mutant has an increased isomerizing activity in comparison to the wild-type protein, while F95Q is impaired in refolding of scRNase.

### 4. The structure of the HP0377-apocytochrome complex is complicated

**Fig 8:**
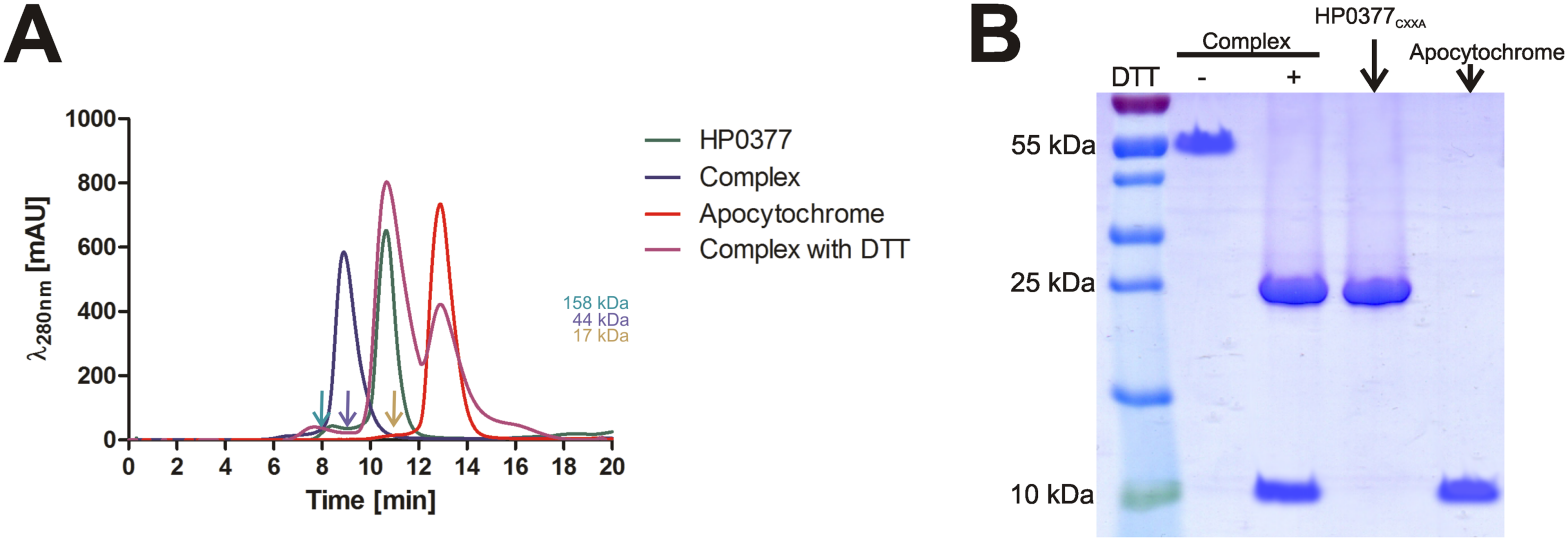
Analysis of HP0377-apocytochrome c complex formation. **(A) Size exclusion profile of the HP0377, apocytochrome, HP0377-apocytochrome complex and HP0377-apocytochrome complex treated with DTT separated on an Enrich**^™^ **sec70 column (Biorad) and monitored by absorbance at 280 nm**. HP0377 elutes as a peak at 10.7 min, with an estimated mass of 23.5 kDa, consistent with the size of the monomer. Apocytochrome elutes at 12.9 min, with estimated mass of 9.5 kDa. Complex eluted at 8.9 min, with estimated mass of 56 kDa. Complex treated with DTT elutes as two peaks at 12.9 min and 10.7 min, consistent with the sizes of the HP0377 and apocytochrome. The column was calibrated with Gel Filtration Standard (Bio-Rad): Thyroglobulin (670 kDa), γ-globulin (158 kDa), Ovalbumin (44 kDa), Myoglobin (17 kDa), Vitamin B12 (1.35 kDa). The relative positions of the chosen standards are marked with arrows. **(B) SDS-PAGE analysis of HP0377-apocytochrome complex formation**. Complexes formed between HP0377_CSYA_ and apocytochrome were first purified using Ni-NTA resin, and then separated on a size exclusion column. The samples were next analyzed by SDS-PAGE with or without DTT.

It was previously documented that replacing the C- terminal cysteine of the CXXC motif of thiol isomerases or reductases with serine or alanine stabilizes their intermediate mixed complexes with substrates, because the C-terminal cysteine of the CXXC motif is indispensable to resolve the intermediate complex [51–54]. Since we noticed that the HP0377 with its CSYC motif changed to CSYS was active in reduction, we decided to use the HP0377 variant with the CSYA motif to analyze its complexing with apocytochrome c. All our attempts to create the complex *in vitro* failed. Thus, we modified the strategy. Lysates of *E*. *coli* strains overexpressing HP0377 and HP1227 were mixed, incubated overnight at 37°C and analysed by size-exclusion chromatography. Using this approach, we succeeded in purification of the stable covalent complex of HP0377_CSYA_ with apocytochrome. As shown in Fig 8A, the overwhelming majority of protein eluted as a peak with an estimated mass of 55 kDa. The separated complex was analyzed by non-reducing (without DTT) and reducing SDS-PAGE. The 55 kDa heterosulfide formed by HP0377_CXXA_ and apocytochrome c disintegrated on the reducing SDS-PAGE into two components with estimated molecular masses of about 23 kDa and 10 kDa, which is equivalent to the molecular masses of HP0377 (lacking signal sequence and with six His residues added to C-terminus) and apocytochrome c, respectively. The data suggest that the complex is composed of two molecules of HP0377 and one apocytochrome c molecule (Fig 8B).

### 5. Phylogenetic analyses

**Fig 9:**
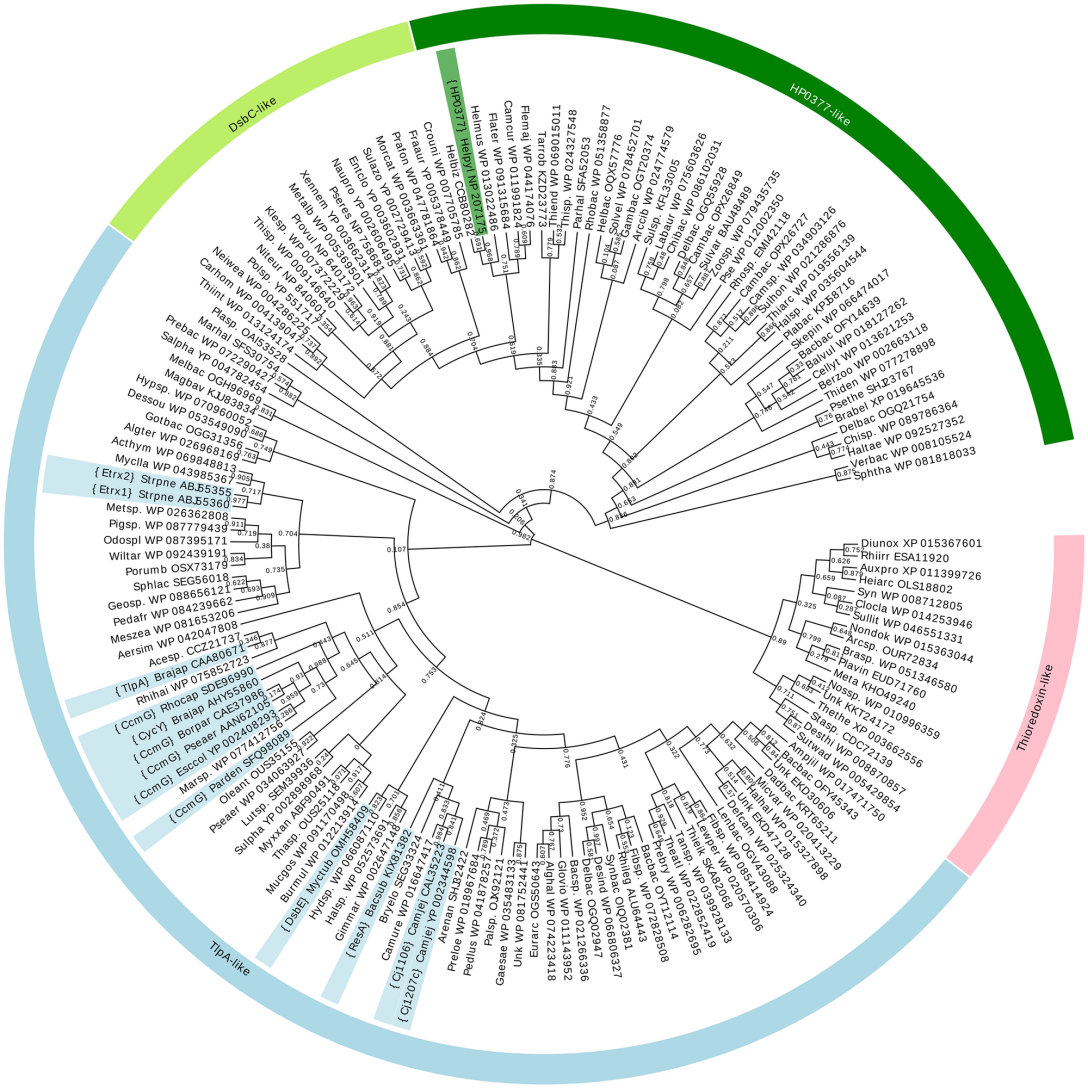
Phylogenetic tree of thioredoxin domains from HP0377 and related proteins. Four clades are indicated on the ring surrounding the tree. Numbers correspond to branch support values provided by the FastTree algorithm. Relevant sequences are highlighted and their names are shown in curly brackets.

To gain insight into the evolution of HP0377 protein, we performed phylogenetic analyses. To this end, we collected >60,000 homologs of the HP0377 thioredoxin domain and clustered them as based on all-vs-all BLAST scores (for details, see Methods). We obtained four separate clusters comprising thioredoxin domains of HP0377-like proteins, DsbC-like proteins, TlpA-like proteins (also contains CcmG-like), and thioredoxin-like proteins. Since the resolution of clustering is too low to infer the relationships between these clusters, we decided to use a classical phylogenetic approach. From each cluster, we selected a set of representative sequences and used them together to calculate a phylogenetic tree (Fig 9). The obtained tree is clearly separated into four statistically well-supported clades corresponding to the four groups identified with the aid of the clustering. Interestingly, the tree revealed that the HP0377-like group is more closely related to the DsbC-like group than to the TlpA/CcmG-like group, implying that HP0377 is not a member of TlpA/CcmG family, but instead is more closely related to DsbC. An analogous evolutionary scenario was observed in the case of HP0231 protein, which despite having DsbA-like activity, is evolutionarily more related to DsbG [25]. The phylogenetic tree shows that proteins such as CcmG, ResA, CycY, Etrx1, Etrx2, and TlpA share common ancestry and are expected to have similar functions. Although HP0377 is capable of complementing ResA in *Bacillus subtilis*, its thioredoxin domain has a different origin, presumably common to DsbC. This is in agreement with the observation that HP0377 is able to refold scRNAse *in vitro* and that its active site CSYC motif resembles the active site of DsbC proteins.

## Discussion

HP0377 thiol oxidoreductase of *H*. *pylori* is a member of the CcmG (*cytochrome c maturation*) family. Previously, we and others showed that HP0377 is able to reduce apocytochrome c in *in vitro* tests. In this work, by introducing *hp0377* into *B*. *subtilis*, we documented for the first time that HP0377 is also involved in the cytochrome biogenesis process *in vivo*. Structures of several representatives of the CcmG family, such as *Bradiryzobium japonicum* (BjCcmG), *Escherichia coli* CcmG (EcCcmG), *Bacillus subtilis* CcmG, (ResA), *Pseudomona aeruginosa* CcmG (PaCcmg), have been solved [12–15]. Additionally, three thiol oxidoreductases of gram-positive bacteria (StoA of *B*. *subtilis* and Etrx1, Etrx2 of *Streptococcus pneumonia)* that are involved in the reduction of disulfide bonds, share several structural and biophysical properties with ResA and are not involved in apocytochrome c reduction, have recently been characterized [55, 56]. Overall, the structure of HP0377 is highly reminiscent of members of the CcmG family, with their typical thioredoxin fold. However, amino acid sequence alignment revealed several differences among HP0377 and other CcmGs, including lack of proline in the CXXC active site, threonine preceding *cis*-proline in the *cis*-proline loop or presence the phenylalanine residue instead of a glutamate residue that is placed three positions after the C-terminal cysteine of the CXXC motive.

The conserved sequence GVXGXPETF (139–147 in EcCcmG) was previously determined as a fingerprint region of the CcmG family [12, 13]. However, this region is not conserved in HP0377 or *B*. *subtilis* ResA (Table 4) Given that CcmGs may interact with three redox partners (DsbD, CcdA—*c**ytochrome* *c d**eficiency* and ScsB–*suppressor of cooper sensitivity*), we hypothesize that the presence of this fingerprint region is restricted to CcmGs being reduced by DsbD or ScsB (Table 4). Stirnimann et al analyzed the structure of nDsb (N-terminated periplasmic domain of DsbD, designated also as the α domain) with EcCcmG and showed that Y141 and E145 are critical for complex formation [22]. ScsB may act in a similar way as DsbD, as these proteins have comparable domain organization (two periplasmic domains–α and γ–connected by β membrane domain with eight transmembrane segments). However, DsbD and ScsB vary substantially in their structure and these differences are clearly visible between their α domains [46]. Nonetheless, as shown in Table 4, CcmGs functioning in bacterial species expressing both, CcdA and ScsBα, possess conserved fingerprinting region, whereas those which express only CcdA do not have this conserved amino acid sequence. The details of the interaction between CcdA, a shorter version of DsbD, with ResA or HP0377 still remain unclear, although a model for transferring electrons by CcdA across the inner membrane has been recently proposed [19, 20]. Previously, we showed that that HP0377 does not interact with EcDsbD [21]. Thus, the interaction between HP0377 and ResA with their redox partner CcdA may use a different mechanism than that displayed by those CcmGs that are reduced by DsbDs. We showed in this paper that *cis*-proline (P156) controls the active site reactivity with redox partner (see below).

HP0377 possesses an active site (CSYC/T*c*P) that resembles the active site of disulfide isomerases such as EcDsbC (CGYC/T*c*P) or CcScsC (CGYC/T*c*P). Presence of Y as the second amino acid of the internal CXXC dipeptide is a hallmark of dimeric thiol oxidoreductases, which act in the isomerization of incorrect disulfides [46, 57]. The next relevant difference between HP0377 and other CcmGs is the presence of phenylalanine located three positions after the C-terminal cysteine residue of the CXXC motif. In contrast, all of the biochemically and structurally characterized CcmGs to date that are involved only in cytochrome maturation, as well as *B*. *subtilis* StoA, possess glutamate in this position. This glutamate has a key role in substrate recognition, at least in the case of ResA and StoA, as ResA (E80Q) and StoA (E71Q) are deficient in their functioning [47, 55]. StoA is an extracytoplasmic, membrane-anchored thiol oxidoreductase with a structure similar to ResA. It is involved in endospore biogenesis, though its direct substrate is unknown [55]. CcmGs possessing glutamate as the third amino acid after the C-terminal cysteine of the CXXC motif also display similarity in the CXXC motif. In all cases, proline is one of the amino acid of the internal dipeptide. In contrast to all of the above-described CcmGs, HP0377 does not have proline as an element of CXXC motif and possesses F in the position of E of other CcmGs. In contrast to E80 of ResA, the F95 of HP0377 has no impact of its reductive activity, though the HP0377 F95Q is impaired in refolding of scRNase. We have concluded that the specific structure of the HP0377 binding cavity, created by interaction of several amino acids, is responsible for its atypical properties and expansion of its substrate specificity, as compared to the majority of CcmGs.

The presence of *cis*-proline, which is adjacent to the CXXC motif in the three dimensional structure but distant in the sequence, is a conserved hallmark of all thiol oxidoreductases. This amino acid residue plays a key role in their interactions with redox partners, as well as with their substrates [58–60]. The HP0377 P156T variant was active *in vitro* in both tests (scRNase refolding and apocytochrome reduction), but its ability to reduce apocytochrome c *in vivo* was significantly reduced, indicating that the 156 *cis*-proline of HP0377 is involved in interaction with redox partner CcdA, rather than substrate recognition. Additionally, its role in the interaction with its downstream partner was confirmed by determining the HP0377 P156T redox state in *B*. *subtilis*. In contrast to HP0377 wild type, which is present in the reduced form, its P156T variant was found in the oxidized state. The data are consistent with observation concerning ResA *B*. *subtilis*, where 141 *cis*-proline plays a role in enzyme stability and reactivity [32]. Also, in the case of EcCcmG, *cis*-proline is important for its reaction with redox partner EcDsbD [13, 32]. However, HP0377 is not re-reduced by EcDsbD. The amino acid preceding the *cis*-proline is characteristic for specific groups of thiol oxidoreductases. Those responsible for disulfide generation nearly always contain valine in this position, whereas threonine is common for isomerases and leucine for cytoplasmic thioredoxins [44]. Most CcmGs possess a small amino acid (alanine, leucine or valine) as the -1 cis-proline amino acid. HP0377, with T*c*P, seems to be an exception. However, the replacement of hydrophilic threonine by hydrophobic valine/leucine does not have an impact on reduction and isomerization protein activity *in vitro*, as well as on the ability to reduce apocytochrome c *in vivo*. The residue preceding the cis-proline P156 of HP0377 is T, and the residue preceding the cis-proline P141 of ResA is L. Those residues are located in the vicinity of the first cysteine from the CXXC motif. It is likely that the residue preceding proline, maintains the proper shape of the cis-Pro loop. According to our analysis, this loop is stabilized differently in the two analyzed proteins. In HP0377 wild type, a hydrogen bond between T155 from the cis-Pro loop and R86 from the C-terminal part of β1 can be observed. In ResA, the L140 residue from the cis-Pro loop interacts with V103 from the C-terminal part of β2. This observation is in agreement with the fact that the T/V mutation of HP0377 does not affect its biological activity. It can, therefore, be supposed that the role of the residue in the position 155 is to maintain the proper shape of *cis*-proline loop via weak hydrophobic interactions with I120. This is in contrast to EcDsbC T182V, which displayed significantly reduced isomerization activity *in vitro* [44].

Our work showed that HP0377 shuffles incorrect disulfide bonds *in vitro* with scRNase as a substrate, and that changing one cysteine residue of the CSYC motif into serine significantly reduced the efficiency of the process, although the mutated HP0377 versions act as reductases towards apocytochrome c, both in *vivo* and *in vitro*. Changing cysteine of the CSYC motif into alanine abolished activity of the protein, both *in vitro* and *in vivo*, confirming the fundamental role of cysteine residues in the process. Also, ResA with cysteine residues of the CXXC motif replaced by alanine is inactive [32]. So, the data raises the question concerning the mechanism of serine action. Serine is an amino acid similar to cysteine in terms of its structure. A side chain consisting of the hydroxyl group in serine is equivalent to the thiol side chain in cysteine. Similarly to the cysteine residue, the serine residue stabilizes the N-terminal cysteine of the CXXC through a hydrogen bond [1, 61]. The mixed complexes between disulfide oxidoreductases and their substrates are short-lived and are difficult to detect. The strategy to identify Dsb targets assume that mutations of the protein’s catalytic site associated with substrate interaction should result in accumulation of mixed intermediate complexes. This hypothesis has been verified for several thiol oxidoreductases. Various mutated versions of the disulfide oxidoreductase were used to stabilize intermediate complexes, and their choice seems to be depend on the role of the protein (oxidase/reductase or isomerase), as well as their structure (monomer vs dimer). In the case of monomeric thioredoxins, variants with CXXS or CXXA motifs are inactive and could be used for substrate identification [53, 62]. In contrast to that, replacing the active proline of the *cis*-proline loop by threonine in monomeric oxidases such as EcDsbA allows intermediate complex accumulation [58, 59]. It should be noted that during the reduction process, the first cysteine of the oxidoreductase CXXC motif is an attacking cysteine and the C-terminal cysteine of the motif is a resolving one, whereas in the case of disulfide generation, this role is carried out by the reduced cysteines of the target protein. In the case of EcDsbC (a dimeric isomerase), its variant with the CXXS motif blocks resolving of complexes with substrate [51, 52] In the case of dimeric bifunctional DsbAs, such as *Legionella pneumophilla* DsbA2 or *Francisella tularensis* DsbA (FliB), variants with CXXA motifs or variants with the proline of the *cis*-proline loop changed to threonine were used. Interestingly, the LpDsbA2 variant with CXXS appeared to be active [63, 64]. In the case of HP0377, analyzed in this paper, both of its variants, one with an SSYC motif and one with a CSYS motif, were able to reduce disulfide bonds of apocytochrome c, but they could not efficiently refold scRNase. The data indicate that HP0377 combines reductive as well as oxidative functions. Consistent with this observation, a CPHS variant of another *H*. *pylori* dimeric oxidoreductase, HP0231, is also not active, as shown by motility tests that are an indicator of protein oxidizing activity [24]. So, the data indicate that changing one cysteine residue of the CSYC into serine results in separation of the two activities of HP0377, reduction and oxidation. We hypothesize that the reduction process is carried out by HP0377 oligomeric forms, with involvement of the second CSYS motif that serves as the resolving cysteine, whereas the oxidizing activity demands the presence of an intact CSYC motif. This hypothesis is confirmed by our data that C92A HP0377 forms a 2:1 complex with apocytochrome and by data presented by Yoon et al. that C92A form 2:2 complex with HP0518 [16]. Additionally, as we found that apocytochrome c can be reduced by HP0377 with a CSYS motif and also by HP0377 with the SSYC motif, we suspect that the HP0377 potentially may undergo some conformational changes and either cysteine of the CSYC motif (N-terminal as well as C-terminal) might function as the attacking amino acid in the reduction process.

## Supporting information

S1 Fig Schematic representation of two selected proteins from the Dsb network. A) *H*. *pylori* (HP0377, PDB: 4FYC), B) *Bacillus subtilis* (ResA, PDB 2G9S). To generate the 2D cartoons shown above, all proteins were divided for three layers (front, middle and back). For each proteins, the thioredoxin fold is bordered with a dotted rectangle.
(TIF)

S2 Fig Superimposition of the secondary structure elements from the thioredoxin fold. *H*. *pylori* (HP0377, PDB: 4FYC)—red, *Bacillus subtilis* (ResA, PDB 2G9S)—green.
(TIF)

S3 Fig Confirmation of *HP0377* production by Western-blot analysis. **(A)** *H*. *pylori* 26695 wt, *B*. *subtilis* wt, *B*. *subtilis* LUL9 and *B*. *subtilis* BS1 proteins (the whole cell lysate) were separated by 12% SDS-PAGE and electrotransferred onto a nitrocellulose membrane. Specific rabbit serum with antibody against HP0377 was used to verify the production of HP0377 in *B*. *subtilis ΔresA* cells. **(B)** *H*. *pylori* 26695 wt, *B*. *subtilis* BS1, *B*. *subtilis* LUL9 and *B*. *subtilis* carrying HP0377-variant proteins (the whole cell lysate) were separated by 12% SDS-PAGE and electrotransferred onto a nitrocellulose membrane. Specific rabbit serum with antibody against HP0377 was used to verify the production of HP0377 in *B*. *subtilis ΔresA* cells.
(TIF)

S4 FigSequence alignment of CcmG proteins.Multiple sequence alignment of HP0377 and related sequences. Consensus secondary structure prediction symbols: alpha-helix: h, beta-strand: e.(DOCX)

S1 Text Additional information concerning S1 and S2 Figs. (DOCX)
