# Supplementary figures and images for: Impact of selected amino acids of HP0377 (Helicobacter pylori thiol oxidoreductase) on its functioning as a CcmG (cytochrome c maturation) protein and Dsb (disulfide bond) isomerase
Source: PLoS One. 2018 Apr 20;13(4):e0195358. doi: 10.1371/journal.pone.0195358 (PMC5909903; doi:10.1371/journal.pone.0195358)

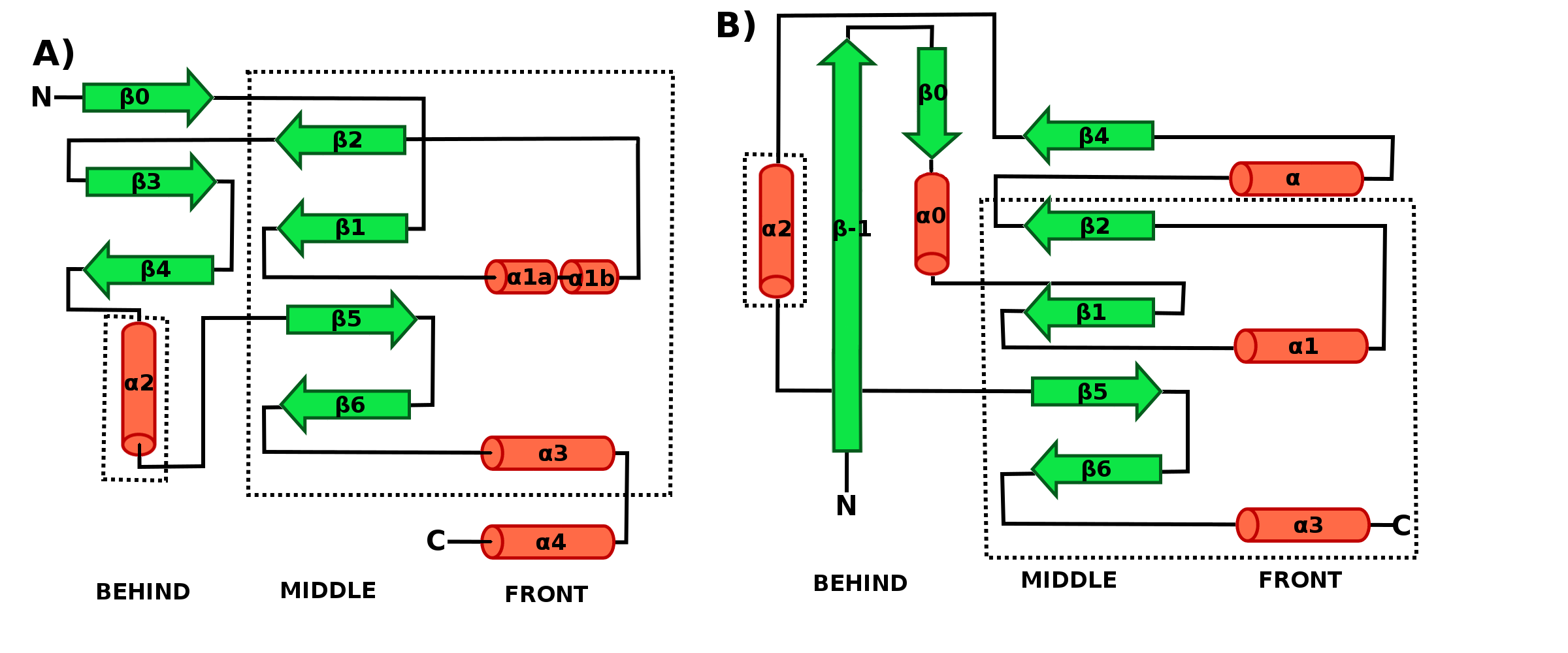

Supplement: S1 Fig — A) H. pylori (HP0377, PDB: 4FYC), B) Bacillus subtilis (ResA, PDB 2G9S). To generate the 2D cartoons shown above, all proteins were divided for three layers (front, middle and back). For each proteins, the thioredoxin fold is bordered with a dotted rectangle. (TIF) [file pone.0195358.s001.tif]

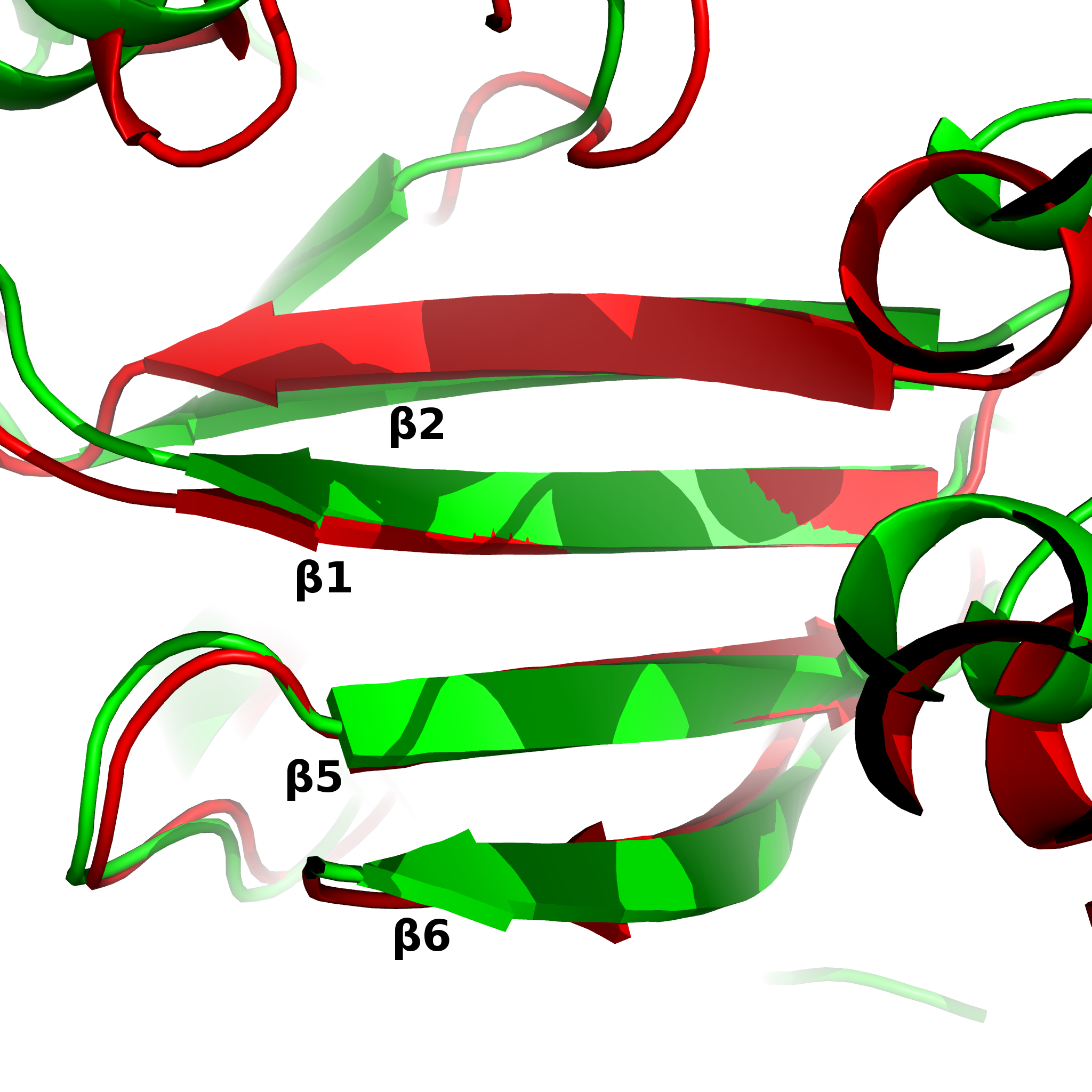

Supplement: S2 Fig — H. pylori (HP0377, PDB: 4FYC)—red, Bacillus subtilis (ResA, PDB 2G9S)—green. (TIF) [file pone.0195358.s002.tif]

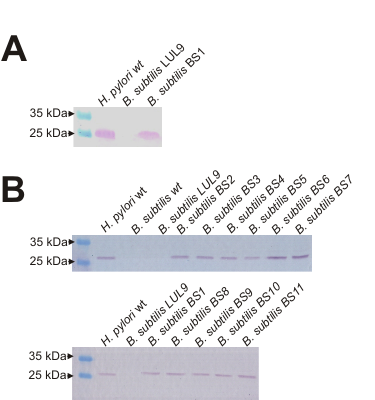

Supplement: S3 Fig — (A) H. pylori 26695 wt, B. subtilis wt, B. subtilis LUL9 and B. subtilis BS1 proteins (the whole cell lysate) were separated by 12% SDS-PAGE and electrotransferred onto a nitrocellulose membrane. Specific rabbit serum with antibody against HP0377 was used to verify the production of HP0377 in B. subtilis ΔresA cells. (B) H. pylori 26695 wt, B. subtilis BS1, B. subtilis LUL9 and B. subtilis carrying HP0377-variant proteins (the whole cell lysate) were separated by 12% SDS-PAGE and electrotransferred onto a nitrocellulose membrane. Specific rabbit serum with antibody against HP0377 was used to verify the production of HP0377 in B. subtilis ΔresA cells. (TIF) [file pone.0195358.s003.tif]
