# Supplementary material for: Impact of selected amino acids of HP0377 (Helicobacter pylori thiol oxidoreductase) on its functioning as a CcmG (cytochrome c maturation) protein and Dsb (disulfide bond) isomerase
Source: PLoS One. 2018 Apr 20;13(4):e0195358. doi: 10.1371/journal.pone.0195358 (PMC5909903; doi:10.1371/journal.pone.0195358)
Supplement: S4 Fig — Multiple sequence alignment of HP0377 and related sequences. Consensus secondary structure prediction symbols: alpha-helix: h, beta-strand: e. (DOCX) [file pone.0195358.s004.docx]

H.pyl_HP0377_NP_207175.1 1 -----MFSLSYVSKKFLSV--L--LLISLFLS---------------ACK-------------SNNKDKL 33

C.jej_Cj1207c_YP_00234459 1 M-----------KIKKILL--L--VAISCLFV------------ACSNDKEKQQ---------NDVNLST 34

M.tub_DsbE_OMH58409.1 1 M-----------RRLVIAAAVS--ALLLTGCSGRDAVAQGGTFEFVSPGG-------------KTDIFYD 44

C.jej_Cj1106_CAL35223.1 1 M-----------KKISALF--L--ISLAFFLN-------------ACSK--------------EEEIQND 28

S.pne_Etrx2_ABJ55355.1 1 M-----------KKVMFAG--L--SLLSLVVL------------MACGEEETKKTQA---AQQPKQQTTV 40

S.pne_Etrx1_ABJ55360.1 1 M-----------KKWQTCV--L--GAGSLLCL------------TACSGKSVTSEHQTKDEMKTEQTASK 43

B.jap_CycY_AHY55860.1 1 MSDQPTSAPPQRRTFLMVLPLI--AFIALALL------------FWFRLG-------------SGDPSRI 43

P.aer_CcmG_AAN62105.1 1 M-----------NRF--LWPLL--GFLVLVSF------------LAVGL--------------TLKPSEV 29

B.par_CcmG_CAE37986.1 1 M-----------LRY--LLPLA--AFLAMAAF------------LALGL--------------GRDPRAL 29

E.col_CcmG_YP002408293.1 1 M-----------KRKVLLIPLI--IFLAIATA------------LLWQLARNAE---------GDDPTNL 36

R.cap_CcmG_SDE96990.1 1 M-----------AKPLMFIPLL--VMAGFVGA------------GFLAMQ-------------KNDPNAM 32

P.den_CcmG_SFQ98089.1 1 M-----------ARFSPMMLLPVAIFAGFAGL------------SGWALL-------------RDDPDAL 34

B.sub_ResA_KIX81382.1 1 M-----------KKKRRLFIRT-GILLVLICA------------LGYTIY-------------NAVFAGK 33

[Consensus_aa:](http://prodata.swmed.edu/promals3d/info/consensus.html) **M**...........p**+**b.*hhh*..*h*...*hh*s*hhh*.............*hh*.s...............p.p....

[Consensus_ss:](http://prodata.swmed.edu/promals3d/info/consensus_ss.html) hhhhhhhhh hhhhhhhh hhh hhh

H.pyl_HP0377_NP_207175.1 34 DENLLSSGSQSSKELNDERDNIDKKSYAGLEDVF----SDNK-SISPNDKYMLLVFGRNGCSYCERFKKD 98

C.jej_Cj1207c_YP_00234459 35 EAS----------INQSDDMNFKLNLIDGG-SISVKKENAVL-NFNDEDKVTLFVFFTTWCTPCIAEIPH 92

M.tub_DsbE_OMH58409.1 45 PPA----------SRGRPGPLSGPELADPAR--------SVS-LDDFPGQVVVVNVWGQWCGPCRAEVSQ 95

C.jej_Cj1106_CAL35223.1 29 FMF----------EEYHKGDKIVLNSVNGGSKTLIRTDKGFV-VEGEEGKVLMFDFFGTFCTPCKEEALD 87

S.pne_Etrx2_ABJ55355.1 41 QQI----------SVGKDVPDFTLQSMDGK---------EVK-LSDFKGKKVYLKFWASWCGPCKKSMPE 90

S.pne_Etrx1_ABJ55360.1 44 TSA----------AKGKEVADFELMGVDGK---------TYR-LSDYKGKKVYLKFWASWCSICLASLPD 93

B.jap_CycY_AHY55860.1 44 PSA----------LIGRPAPQTTLPPLEGLQADNS-QVPGLD-PAAFKGKVSLVNVWASWCVPCHDEAPL 101

P.aer_CcmG_AAN62105.1 30 PSP----------LINKPAPRFSLPQLQAPD-------LTFS-SESMIGKVWVLNVWASWCAPCLEEHPV 81

B.par_CcmG_CAE37986.1 30 PSA----------MLDKPAPPIGLPLLQGDG-------RRLE-VAQLRGRVWVLNVWASWCAPCREELPL 81

E.col_CcmG_YP002408293.1 37 ESA----------LIGKPVPKFRLESLDNPG-------QFYQADVLTQGKPVLLNVWATWCPTCRAEHQY 89

R.cap_CcmG_SDE96990.1 33 PTA----------LAGKEAPAVRLEPLGAEV-------PFTD-ADLRDGKIKLVNFWASWCAPCRVEHPN 84

P.den_CcmG_SFQ98089.1 35 PSA----------MIGREAPSVGEATLPGKV-------QLTD-EMLRQPGVKLVNFWASWCPPCRAEHPT 86

B.sub_ResA_KIX81382.1 34 ESI----------SEGSDAPNFVLEDTNGK---------RIE-LSDLKGKGVFLNFWGTWCEPCKKEFPY 83

[Consensus_aa:](http://prodata.swmed.edu/promals3d/info/consensus.html) ...............sc.ss.*h*.b..*h*st...........*h*p.....ps**+**.*hhl*s*h@*to*@***C**ss**C**..p*h*..

[Consensus_ss:](http://prodata.swmed.edu/promals3d/info/consensus_ss.html) eee ee hhhh eeeeeee hhhhhhhhh

H.pyl_HP0377_NP_207175.1 99 LKNVKELRDYIK-EHFSAYYVNISY-----SKEHDFKVGDKNNEK---EIKMSTE-ELAQIY-------A 151

C.jej_Cj1207c_YP_00234459 93 LN---KLQEKYN-NDFNIVGVLLED----KSNDEIQKFIEQHKISYKVANGENNYLLAKALG-------G 147

M.tub_DsbE_OMH58409.1 96 LQ---RVYDATRGAGVSFLGIDVRD----NNRQAPQDFINDRHVTYP-SIYDPAM-RTLIAFGG---KYP 153

C.jej_Cj1106_CAL35223.1 88 LS---KLWKNNS-SKFIIIGLTHFED---VSDETVKKFAGDYGAYYFLSNGSSND-RIIAQILKDIDYQN 149

S.pne_Etrx2_ABJ55355.1 91 LM---ELAAKPD-RDFEILTVIAPGIQGEKTVEQFPQWFQEQGYKDIPVLYDTKA-TTFQAY-------Q 148

S.pne_Etrx1_ABJ55360.1 94 TD---EIAKEAGDDYVVLTVVSPGHKG-EQSEADFKNWYKGLDYKNLPVLVDPSG-KLLETY-------G 151

B.jap_CycY_AHY55860.1 102 LT---ELAKDK---RFQLVGINYKD-----AADNARRFLGRYGNPFGHVGVDANG-RASIEW-------G 152

P.aer_CcmG_AAN62105.1 82 IT---ELASRH---AVSVVGMNYKD-----TPQNAIAWLRRNGNAFETTVSDAHG-AVGIDF-------G 132

B.par_CcmG_CAE37986.1 82 LQ---EVGPRD---AVPIYGLNYKD-----KPEDARAWLARHGNPYVASASDVDG-RVGIEY-------G 132

E.col_CcmG_YP002408293.1 90 LN---QLSAQ----GIRVVGMNYKD-----DRQKAISWLKELGNPYALSLFDGDG-MLGLDL-------G 139

R.cap_CcmG_SDE96990.1 85 LI---ALKQD----GFEIMGVNWKD-----TPDKAQGFLAEMGSPYTRLGADPGN-RMGLDW-------G 134

P.den_CcmG_SFQ98089.1 87 LT---ELSAR-----LPVYGVDLKD-----PEGAALGFLSEHGDPFHALAADPRG-RVAIDW-------G 135

B.sub_ResA_KIX81382.1 84 MA---NQYKHFKSQGVEIVAVNVGE-----SKIAVHNFMKSYGVNFP-VVLDTDR-QVLDAY-------D 136

[Consensus_aa:](http://prodata.swmed.edu/promals3d/info/consensus.html) *l*....p*l*..p.....*h*.*lh*s*l*s*h*.c.....s...*h*..*@h*.p.s..*@*.....ssp...*h*...*h*.......s

[Consensus_ss:](http://prodata.swmed.edu/promals3d/info/consensus_ss.html) hh hhhhh eeeeee hhhhhhhhhh eeee h hhhhh

H.pyl_HP0377_NP_207175.1 152 VQSTPT-----------IVLSDKTGKTIYELPGYMPSTQFLAVLEFIGDGKYQDTKDDEDLTKKLKAYIK 210

C.jej_Cj1207c_YP_00234459 148 VNGIPT-----------MFLYNKHSKLINQYLGLIPEEMLEIDIQKAIL--------------------- 185

M.tub_DsbE_OMH58409.1 154 TSVIPS-----------TLVLDRQHRVAAVFLRELLAADLQPVVERVAEEEPSGRAPVGAQ--------- 203

C.jej_Cj1106_CAL35223.1 150 MEQLPFKVVLKNGIYQKISDYWNNNTPTNFYLGKIPTELMQEDLNKIYKGK------------------- 200

S.pne_Etrx2_ABJ55355.1 149 IRSIPT-----------EYLIDSQGKIGKIQFGAISNADAEAAFKEMN---------------------- 185

S.pne_Etrx1_ABJ55360.1 152 VRSYPT-----------QAFIDKEGKLVKTHPGFMEKDAILQTLKELS---------------------- 188

B.jap_CycY_AHY55860.1 153 VYGVPE-----------TFVVGREGTIVYKLVGPITPDNLRTVLLPQMEKALKAGS-------------- 197

P.aer_CcmG_AAN62105.1 133 VYGVPE-----------TYVIDKAGIIRYKHTGAIDAGEMRGELLPLVRELEK----------------- 174

B.par_CcmG_CAE37986.1 133 VYGVPE-----------TFVIDGAGRIRYRQLGVLTPQIWRERLLPVIEGLR------------------ 173

E.col_CcmG_YP002408293.1 140 VYGAPE-----------TFLIDGNGIIRYRHAGDLNPRVWEEEIKPLWEKYSKEAAQ------------- 185

R.cap_CcmG_SDE96990.1 135 VAGVPE-----------TFVVDGTGRILTRIAGPLTEDVITRKIDPLLAGPAE----------------- 176

P.den_CcmG_SFQ98089.1 136 VTAPPE-----------TFIIDGSGRILHRHAGPLVREDYTNRFLPELEKALAAE--------------- 179

B.sub_ResA_KIX81382.1 137 VSPLPT-----------TFLINPEGKVVKVVTGTMTESMIHDYMNLIKPGETSG---------------- 179

[Consensus_aa:](http://prodata.swmed.edu/promals3d/info/consensus.html) *l*.s*h***P**p...........*hhlh*s.ptp*h*...*hh***G**.*h*s...*h*p..*h*..*h*.......................

[Consensus_ss:](http://prodata.swmed.edu/promals3d/info/consensus_ss.html) e eeee eeeeeee hhhhhhhhhhhhhhh

H.pyl_HP0377_NP_207175.1 211 YKTNLSKSKSN 221

C.jej_Cj1207c_YP_00234459 -----------

M.tub_DsbE_OMH58409.1 -----------

C.jej_Cj1106_CAL35223.1 -----------

S.pne_Etrx2_ABJ55355.1 -----------

S.pne_Etrx1_ABJ55360.1 -----------

B.jap_CycY_AHY55860.1 -----------

P.aer_CcmG_AAN62105.1 -----------

B.par_CcmG_CAE37986.1 -----------

E.col_CcmG_YP002408293.1 -----------

R.cap_CcmG_SDE96990.1 -----------

P.den_CcmG_SFQ98089.1 -----------

B.sub_ResA_KIX81382.1 -----------

[Consensus_aa:](http://prodata.swmed.edu/promals3d/info/consensus.html) ...........

[Consensus_ss:](http://prodata.swmed.edu/promals3d/info/consensus_ss.html)
