# Supplementary material for: Impact of selected amino acids of HP0377 (Helicobacter pylori thiol oxidoreductase) on its functioning as a CcmG (cytochrome c maturation) protein and Dsb (disulfide bond) isomerase
Source: PLoS One. 2018 Apr 20;13(4):e0195358. doi: 10.1371/journal.pone.0195358 (PMC5909903; doi:10.1371/journal.pone.0195358)
Supplement: S1 Text — (DOCX) [file pone.0195358.s005.docx]

The folds of HP0377 and ResA are schematically depicted in **S1** and **S2 Figs**. It can be seen from the **S1** **Fig** that both selected proteins from the Dsb network show very high structural similarity of the β1, β2, β5 and β6 strands from the thioredoxin fold, with the RMSD less than 1Å from each other.

For *H. pylori* (HP0377, PDB: 4FYC), the α1 helix is divided into two segments denoted as α1a and α1b, respectively. These fragments are connected by N101 and V102 residues. According to statistical analysis of protein structures [1], residues of these two kinds are located in β strands and turns. In both analyzed proteins (HP0377 and ResA), a highly conservative C92 residue from the C89XXC92 motif is located in the N-terminal part of α1. This cysteine residue interacts with the highly conserved P156 residue from the cis-Pro-loop, which is located in the N-terminal part of the β5 strand. The rest of the α1 - β5 interactions are different; however, it seems that they stabilize the fold. For example, in HP0337 from *H. pylori*, one helix turn later in the sequence, there is an F95 (α1) – I158 (β5) hydrophobic interaction, which is replaced by an E80 (α1) – T143 (β5) hydrogen-bonding interaction in ResA from *B. subtilis*. In both proteins, the α3 helix is located in the vicinity of the β1, β5 and β6 strands; however the helix in ResA is shifted along the β-strands with respect to that in HP0377. As a consequence, the α3 helical sections from the two species do not superimpose well on each other.

In HP0377, α3 interacts with β5 and β6 while, in ResA, the position of α3 is shifted towards β1. As a consequence, additional interactions of α3 with this fragment can be observed. The α2 helix is located on the other side of the four-stranded β-sheet compared to α1 and α3 and interacts with β1, β2 and β5 mostly via hydrophobic interactions.

The biggest differences occur in the N-terminal segment. For the protein from *H. pylori*, the 55 residues from the N-terminal part of the protein are not present in the crystal structure; however, an analysis of the HP0377 structure shows that the β1 strand is connected to the β0 strand. For ResA, an additional small helix (α0) can be observed in this region. In the HP0377 structure, β1 is missing; however, β3 and β4, forming an anti-parallel β-sheet, occur in this region. The cis-Pro loop connects α2 and β5 from the thioredoxin fold, and it has an almost identical structure in both HP0377 and ResA, with a hydrophobic residue located in the middle, which interacts with β6 and creates a bend. Additionally, an identical hydrophobic interaction of V152/Vl137 (cis-proline loop) with F84/E69 (β1) from HP0377/ResA, respectively, can be found.

As was pointed out, the proline residue is located in the C-terminal part of cis-proline loop, and in both proteins, it interacts with the two cysteines from the CXXC motif located in the N-terminal part of α1.

1. Creighton TE. Proteins: Structures and Molecular Properties. Freeman W. H.; 1992.
